# Social determinants of access to reproductive health services among Afghan migrant women in Iran: A qualitative study

**DOI:** 10.1371/journal.pgph.0007406

**Published:** 2026-10-15

**Authors:** Seyedeh Tahereh Mirmolaei, Zohreh Mahmoodi, Amirhossein Takian, Zahra Bayat Jozani

**Affiliations:** 1 Department of Midwifery and Reproductive Health, School of Nursing and Midwifery, Tehran University of Medical Sciences, Tehran, Iran; 2 Social Determinants of Health Research Center, Alborz University of Medical Sciences, Karaj, Iran; 3 Department of Health Management, Policy and Economics, School of Public Health, Tehran University of Medical Sciences, Tehran, Iran; 4 Health Equity Research Centre (HERC), Tehran University of Medical Sciences, Tehran, Iran; 5 Centre of Excellence for Global Health (CEGH), Department of Global Health and Public Policy, School of Public Health, Tehran University of Medical Sciences, Tehran, Iran; Islamic Azad University South Tehran Branch, IRAN, ISLAMIC REPUBLIC OF

## Abstract

Migrant women, particularly those from low-resource settings, often face restricted access to reproductive health services in host countries due to intersecting social, economic, and structural barriers. This study explored these barriers among Afghan migrant women in Iran using the World Health Organization's Social Determinants of Health (SDH) model. This qualitative study employed semi-structured, in-depth interviews with 20 purposively sampled Afghan migrant women of reproductive age and 10 key informants from healthcare, midwifery, NGO, and policy backgrounds**.** Maximum variation sampling was used to ensure diversity in residency status, socioeconomic background, and service experiences. Data collection took place in Tehran from April 2024 over six months and analyzed using directed content analysis guided by the World Health Organization's Social Determinants of Health (SDH) framework. Prolonged member checking and peer debriefing enhanced the credibility and confirmability of the final categorization of structural and intermediary determinants. Three interconnected levels of determinants shape Afghan migrant women’s reproductive health access. Structural determinants include a restrictive legal and macroeconomic context, undocumented status, banking and communication barriers, and high out-of-pocket costs, compounded by patriarchal norms that limit women’s agency. Intermediary determinants include poor material circumstances, psychosocial stressors, and bio-behavioral responses, with care-seeking delays reflecting legal insecurity. Health system and cross-cutting factors, including the lack of migrant-sensitive facilities, linguistic mismatches, and perceived discrimination, further deter service utilization. Social capital and NGO support provide vital bridges to care, yet the intersection of these determinants creates a vulnerability that perpetuates health inequities and restricts access to essential services. This study clarifies how structural and intermediary determinants interact to shape the barriers Afghan migrant women face in accessing reproductive health services in Iran. These findings can inform targeted, migrant-sensitive policy and programmatic interventions to enhance service equity and lower long-term healthcare costs for both migrant populations and the host country.

## Introduction

Migration is a complex and multidimensional social phenomenon that significantly affects the health and well-being of both migrant populations and host communities [1]. Due to its geographical proximity to Afghanistan and the ongoing economic, security, and political instability in the region, Iran has hosted successive waves of Afghan migration over recent decades and is now one of the largest refugee-hosting countries in the world. Official figures indicate approximately 3.47 million registered Afghan nationals, while recent estimates, including undocumented individuals and those who arrived after the 2021 political transition, place the total population at over 4.5 million [2,3].

In this context, it is essential to distinguish between migrants, individuals who primarily move for economic or educational reasons, and refugees, those who flee due to a well-founded fear of persecution or conflict [3]. In Iran, this distinction has direct implications for legal status and access to services. Registered refugees (mainly Amayesh cardholders) generally have structured access to public health services and insurance, whereas undocumented migrants face severe structural barriers, ranging from the constant fear of deportation to near-complete exclusion from formal healthcare facilities [4,5].

Sexual and Reproductive Health (SRH) is more than a fundamental human right; it is one of the cornerstones of the Sustainable Development Goals and directly influences the health trajectories of individuals and society [6]. According to Levesque et al. [7], “access” to healthcare refers to the opportunity for individuals to reach, obtain, and utilize appropriate services when they perceive a need for care. This includes five core dimensions: approachability, availability and accommodation, affordability, acceptability, and appropriateness [7].

For Afghan migrant women in Iran, SRH status is shaped by a complex intersection of socio-demographic, cultural, and structural factors. Iranian law — including constitutional provisions on the right to health and social security (Articles 29 and 43) and the mandatory insurance clause for foreign nationals under the Sixth Five-Year Development Plan (Article 70) — has been interpreted as establishing a legal commitment to provide primary healthcare, including maternal and child health services, to Afghans regardless of legal status. However, a significant gap persists between this legal entitlement and actual utilization [5]. The Iranian context creates a distinct pattern of health inequity driven by restrictive migration policies, economic sanctions, legal invisibility, and patriarchal norms. Applying a gender and intersectional lens reveals how legal precarity, economic vulnerability, low education, and gender inequality intersect to produce compounded disadvantages, particularly for undocumented and low-educated women. These socially patterned inequalities threaten not only individual health but also broader public health goals, including maternal mortality reduction and health equity [8–10].

To analyze these dynamics, the present study uses the World Health Organization’s Social Determinants of Health (SDH) framework [11]. This framework distinguishes between structural determinants and intermediary; while highlighting the central role of social cohesion and capital (Fig 1) and this study analyzes the five dimensions of access through the WHO-SDH framework. Structural determinants (governance, legal status, and norms) primarily shape approachability, affordability, and acceptability; for example, restrictive policies and poverty diminish access via deportation fears and lack of insurance. Additionally, intermediary determinants (material conditions and health system factors) impact availability and appropriateness. Barriers such as distance, language gaps, and discrimination dictate the practical adequacy of care, effectively mediating the relationship between macro-structures and women's actual SRH access [7,11].

**Fig 1:**
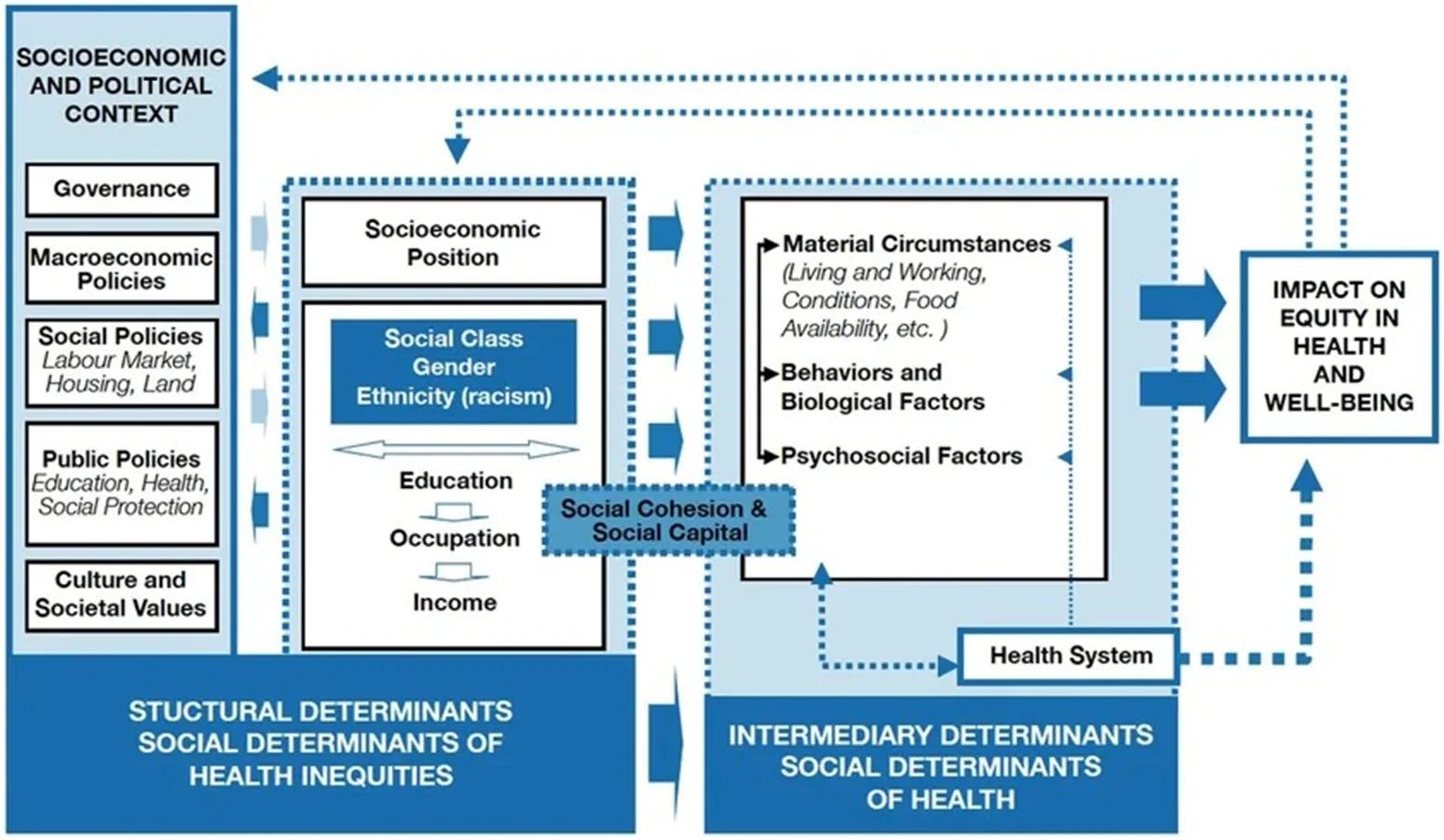
WHO framework of social determinants of health [11].

By moving beyond clinical care, this framework addresses health inequities shaped by power relations and broader life circumstances [10,12]. Systemic obstacles, ranging from limited health literacy to discrimination and financial strain, collectively heighten maternal mortality risks for migrant women [13]. Despite previous efforts, many studies lack a comprehensive framework integrating pathways like intersectionality [5,13] and rely on regression models designed for high-income settings (HICs). Such models often fail to encapsulate the unique structural determinants of LMICs like Iran, including the legal invisibility of undocumented migrants and economic volatility from sanctions. Consequently, there is a critical gap in applying the WHO-SDH framework to analyze SRH access in these complex host environments [12,13].

To date, no study in Iran has utilized the SDH framework to examine the social determinants influencing Afghan migrant women’s access to reproductive health services. This study aimed to illuminate the social determinants of health influencing SRH access among Afghan migrant women in Iran using an intersectionality lens. By exploring their lived experiences, the study seeks to provide actionable insights for addressing unmet needs and promoting health equity in reproductive health services (Fig 1).

## Methodology

### Ethics statement

This study was approved by the Research Ethics Committee of the Faculty of Nursing and Midwifery, Tehran University of Medical Sciences (approval number IR.TUMS.FNM.REC.1402.090). All participants, including Afghan migrant women and key informants, received information about the study's aims and procedures. Written informed consent was obtained from all participants before the interviews. Participation was voluntary, and participants were told they could withdraw at any time. Confidentiality and anonymity were ensured by de-identifying all interview data.

This qualitative study was conducted as part of a doctoral thesis and employed directed qualitative content analysis [14] guided by the WHO’s (SDH) framework [11]. The analytical process followed the 16-step procedure proposed by Assarroudi et al. [15]. To ensure methodological transparency and rigor, the study was reported according to the Consolidated Criteria for Reporting Qualitative Research (COREQ) [16]. A completed COREQ checklist is provided as S1 Checklist.

### Study design and sampling

A qualitative study using purposive sampling with maximum variation was conducted to capture diverse experiences among Afghan migrant women. Inclusion criteria comprised women aged 18–49 who had resided in Tehran for at least one year, possessed the ability to converse in Persian or Dari, and had no cognitive or mental disabilities that would impede communication. To operationalize an intersectionality lens, we intentionally sought diversity across residency status (Amayesh Card, or undocumented) and its intersection with socioeconomic backgrounds and education levels. This allowed for an exploration of how overlapping identities create unique barriers to SRH services. Recruitment occurred at health centers, municipal recreational hubs, and educational centers to include non-users of formal services. Additionally, 10 key informants were interviewed.Sampling continued until data saturation was achieved for both participant groups. Among the 20 Afghan migrant women, code saturation—the point at which no new codes emerged—was reached after the 17th interview, with three subsequent interviews conducted to confirm meaning saturation, i.e., that no further conceptual depth or variation emerged within the existing codes. Among the 10 key informants, code saturation was reached after the 8th interview, with two additional interviews conducted for the same confirmatory purpose. Coding was performed independently by two coders—the corresponding author as first coder and the first author as second coder—and saturation was jointly confirmed through discussion and consensus with the wider research team [17,18]. Semi-structured interview guides were used for Afghan migrant women and key informants (S1 Text). This study is reported in accordance with the Consolidated Criteria for Reporting Qualitative Research (COREQ) (S1 Checklist).

### Data collection

From April 2024 to June 2025, the lead researcher conducted semi-structured, in-depth interviews (45–60 minutes) in locations ensuring participant comfort, including telephone options. The interviews were conducted using a semi-structured guide developed based on the WHO-SDH framework (S1 Text). Interviews were audio-recorded with consent and supplemented by field notes. To ensure a more inclusive sample and to reach women who might not regularly utilize formal health services, recruitment was expanded beyond health centers to include municipal educational and recreational hubs (specifically ‘Shahr-banoo’ centers dedicated to women) and local community centers and Nongovernmental organization. This strategy allowed us to capture the perspectives of ‘non-users’ and those living in extreme legal or social invisibility.

### Data processing and analysis

To bridge cultural and linguistic gaps, an Afghan midwifery student served as a peer facilitator to build trust and ensure accurate interpretation. Transcriptions were analyzed via directed qualitative content analysis using an *a priori* codebook derived from the WHO-SDH framework. The 16-step procedure by Assarroudi et al. [15] was followed, integrating both deductive and inductive coding.

### Trustworthiness

According to Lincoln and Guba’s criteria [19], trustworthiness was established as follows. Credibility was ensured through prolonged engagement in the field, member checking with 10 of the 30 participants who reviewed transcripts of their own interviews, and team debriefing sessions held biweekly throughout the analytic period. Dependability was maintained via a detailed audit trail comprising field notes, an evolving codebook, and successive versions of the coding scheme. Confirmability was addressed through a reflexive journal maintained by the corresponding author across six months. Thick descriptions of the context and participants are provided in the Findings section to enhance transferability.

### Research team and reflexivity

The lead researcher's professional background as a midwife facilitated a higher level of trust and ‘linguistic comfort’ during interviews, particularly regarding sensitive sexual and reproductive health (SRH) topics. However, it is important to note that the researcher did not engage with participants in a clinical capacity at any point during data collection; her role throughout the interviews was strictly that of a researcher, not a healthcare provider. This clinical background nonetheless carried a potential risk of bias, namely a tendency to interpret participants’ narratives through a medicalized lens rather than remaining fully open to their own framing of their experiences. To mitigate this risk, transcripts and preliminary codes were reviewed by a non-clinical member of the research team, and discrepancies in interpretation were resolved through team discussion. This reflexive process allowed the research team to draw on the researcher's clinical familiarity as an asset for rapport-building while guarding against its potential to narrow the interpretive lens.

### Findings

The socio-demographic profile of the 20 participants (45% documented, 55% undocumented) revealed significant disparities based on residency status. While the groups were similar in age, undocumented women reported a lower mean age at marriage (16.8 years), higher parity (median: 2), and substantially higher rates of illiteracy among both themselves (55%) and their husbands (55%) compared to their documented counterparts. Duration of stay varied widely, with a median of 4.5 years. Detailed characteristics are presented in Table 1.

**Table 1:** Summary of key characteristics of participating Afghan migrant women by residency status (N = 20).

Characteristic	Documented (n = 9)	Undocumented (n = 11)	Total (N = 20)
Age (years), mean (range)	28.8 (21–45)	28.5 (22–44)	28.6 (21–45)
Age at marriage (years), mean	18.8	16.8	17.7
Duration of stay in Iran (years), median (range)	5.0 (1.2–33)	3.0 (1.5–16)	4.5 (1.2–33)
Education (years), median	6.0	7.0	6.0
No formal education / illiterate, n (%)	1 (11%)	6 (55%)	7 (35%)
Number of children, median	0	2	0
Husband with no formal education, n (%)	2 (22%)	6 (55%)	8 (40%)
Husband’s age (years), mean	36.7	36.2	36.4
Ethnic background, Hazara, n (%)	9 (53%)	8 (47%)	17
Pashtun, n (%)	2(66%)	1(33%)	3

The 10 key informants (KIs) represented a diverse group of experts, including midwives, clinicians, academic instructors, NGO representatives, and policymakers. Collectively, they provided extensive professional expertise, averaging 15 years (range: 5–30 years) of direct involvement in clinical care, education, and policy development for migrant populations in Tehran. Their multidisciplinary backgrounds ensured a comprehensive understanding of the systemic and structural barriers impacting Afghan women's reproductive health. Detailed profiles of the informants are presented in Table 2.

**Table 2:** Characteristics of key informants interviewed (N = 10).

Participant ID	Type of organization / setting	Years of relevant experience	Main role / type of services provided
KI-01	NGO focused on harm reduction and support for vulnerable populations	Since establishment (≈15 years)	Support and services for homeless and substance-affected women and men
KI-02	University-affiliated public health department (reproductive health unit)	8	Reproductive health policy, service delivery, statistics, and program evaluation
KI-03	Professional midwifery association; university faculty; former policy maker	30	Policy development, clinical midwifery services, and education
KI-04	Public maternity hospital (government)	15	Clinical obstetric and midwifery services in delivery ward
KI-05	Public health center (urban)	10	Clinical midwifery care and reproductive health counseling
KI-06	Medical council office + public health base + ministerial experience	15	Education, clinical services, and policy-related activities
KI-07	Private midwifery counseling center; university instructor; hospital services	20	Clinical care, midwifery education, and private counseling
KI-08	University counseling center + NGO activities	23	Psychosocial counseling and social support services
KI-09	Public health center; university midwifery instructor	8	Clinical reproductive health services, counseling, and training
KI-10	Public maternity hospital (government); PhD in reproductive health	5	Clinical midwifery services in labor and delivery ward

Interview findings were systematically synthesized and categorized according to the WHO Social Determinants of Health framework. This analysis produced a structured hierarchy comprising overarching themes, categories, subcategories, and final codes. The identified factors influencing the access to and utilization of reproductive health services are detailed in Table 3.

**Table 3:** Factors affecting access to and use of reproductive health services based on the World Health Organization model extracted from interviews.

Main Category	Sub-category	Final Codes
**1. Structural Determinants**	Governance & Legal Status	1- Impact of strict migration policies and residency status 2- Regulations on property, banking, and mobile registration
	Macroeconomic Policy	1- Economic challenges of the host country 2-Legal employment restrictions
	Social Policy	1-- Housing policy 2- Equal access to insurance and social safety nets 3- Employment regulations for migrants
	Public Policy	1- Collaboration with international organizations and NGOs **2-** Educational, digital-literacy, and media awareness campaigns for migrants 3- Absence of subsidized health insurance / free health services for migrants 4- Absence of low-cost or social housing support for migrant families 5- Legal restrictions on specific SRH services (e.g., contraception, safe abortion), forcing reliance on unlicensed providers 6- Registration without functional follow-through (‘register and wait’) and lack of affordable structural alternatives
	Cultural & Social Values	1- SRH-related taboos and traditional beliefs 2 - Preference for home births 3- Stigma and discrimination 4- Patriarchal norms and gender inequality
	Social class	1-Family income and financial adequacy 2-Education of migrants and her family 3- Employment/Occupation
**2. Intermediary Determinants**	Material Circumstances	1- Housing quality and household density 2- Job instability and unsafe work 3- Nutrition and food security
	Psychosocial Factors	1- Migration-related stress and anxiety 2- Fear of arrest and living secretly 3- Fear of spousal abandonment 4- partner violence
	Bio-behavioral Factors	1- Medical history and perceived need for SRH services 2- Delay in seeking care due to deportation fears
	Accessibility & Health Service Delivery	1- Financial Access: High cost of health services 2- Geographical Access: Distance to health centers 3- Service Availability: Access to mobile clinics and public health programs 4- Facility Sensitivity: Lack of migrant-sensitive facilities
	Quality Health Service & Cultural Safety	**1-** Patient-Centeredness / Responsiveness: Inequality and discrimination in service provision 2- Cultural Safety / Effectiveness : Lack of cultural and linguistic competence among staff
**3. Cross-cutting Factors (Mediating**)	Social Cohesion & Social Capital	1- Trust in the healthcare system 2- Social participation 3-acceptance by host society
	Social Support Networks	1. Support from family and spouse 2. Support from NGOs and peer network

The qualitative analysis identified key structural, intermediary, and cross-cutting determinants affecting Afghan migrant women’s access to reproductive health services. These findings are presented according to the adapted WHO Social Determinants of Health framework.

## 1. Structural determinants of health

### Governance and legal status

Legal status was one of the most prominent barriers. Many women avoided healthcare facilities due to lack of official documents or fear of deportation. Participant 5, an undocumented migrant, said: “*To buy things or see a doctor or get treatment, we need a bank card, which they don't give us. So, we bought one from a neighbor under his wife's name and we pay her rent monthly*.”

Participant 6, who needed an ultrasound due to vaginal bleeding, explained: *“I needed an ultrasound, but they asked for insurance and identification. I had neither, so they refused to do the test*.”

### Macroeconomic policy

Economic challenges and legal employment restrictions significantly limited access to services. Participant 20 described the situation as follows: *“What I am doing now is because of the bad conditions. I wish I could work at a clinic or help my fellow countrymen somewhere, like at a charity center. Afghans are forced to do illegal work without insurance because we don't have permission to work.*”

### Social policies

Social policy emerged as a key structural determinant influencing access to reproductive health services. Participants frequently highlighted the fragility of welfare policies, particularly in the areas of housing, insurance coverage, and employment regulations. Housing instability was a major concern. Participant 19, who faced significant challenges with housing and nutrition, explained how basic survival needs took priority: “*We were more concerned about work, food, and shelter; we didn't have money for a doctor. If I had to spend money on a doctor, I would rather spend it on the kids.*” Lack of equal access to insurance and social safety nets was repeatedly mentioned as a barrier. Participant 6 stated: “*I couldn't get medications and medical services like ultrasound because I don't have an ID card or insurance.”* Employment regulations for migrants further restricted access. Many women noted that legal limitations on formal employment left their families without stable income or insurance, making healthcare costs difficult to afford.

### Public policies

Lack of information and education about reproductive health services was frequently reported as a barrier. Participant 1 suggested the need for awareness campaigns, stating: “*Many teenage girls during menstruation are considered impure, do not go to sacred places, and even lack sanitary pads.*” Participant 6 highlighted the absence of health insurance and high costs: “*I couldn't get medications and medical services like ultrasound because I don't have an ID card or insurance*.” Participant 15 emphasized a lack of awareness about available services: “*No, I didn't* *go to any of the screenings. I didn't know I had to go; no one told me*.” The midwife in charge of the maternity ward noted low digital literacy as a critical barrier: “*The level of digital literacy among Afghan migrants is lower, and they do not seek services because many of them do not even know what health services are available or how they can access them*.”

Beyond awareness gaps, participants repeatedly described the absence of concrete structural policy support for migrants. Regarding health-insurance subsidies, Participant 7 explained: “*I came for a pregnancy checkup, but there is no free health center near us. They told me I don’t have insurance and have to pay cash. Where would I get that from? My husband is a day laborer; he can barely bring home bread for dinner.*”

Legal restrictions on specific SRH services further pushed women toward unlicensed care. Participant 4 stated: “*Reproductive health services? They exist only in name. For birth control pills or an abortion, I have to go to unlicensed clinics — they say they’re not allowed, that the country doesn’t permit it. There is no one to sort things out for me in one place.*”

Even where formal registration existed, participants described it as procedural rather than functional. Participant 17 explained: “*There is no support here for an Afghan woman. They tell you to go back to your country, or come legally, or go to the private sector — but that’s beyond what we can afford too. They only register you and tell you to wait.*”

### Cultural and social values

Cultural beliefs and traditions strongly influenced health-seeking behavior. Sexual and reproductive health topics were often considered taboo. Many women preferred home births due to cultural customs. A private midwife noted: “*Many of our clients want home births. We assess the risk and provide prenatal education*.” Participant 3 shared: “*I had two deliveries in the hospital and two at home.*” Low literacy was another challenge. Participant 2 said: “*It would be much better if doctors could give lectures or if there were videos available on the internet*.”

### Cultural and social values

Cultural beliefs and traditions strongly influenced health-seeking behavior. Sexual and reproductive health topics were often considered taboo. Many women preferred home births due to cultural customs. A private midwife noted: “*Many of our clients want home births. We assess the risk and provide prenatal education*.” Participant 3 shared: “*I* *had two deliveries in the hospital and two at home.*” Low literacy was another challenge. Participant 2 said: “*It would be much better if doctors could give lectures or if there were videos available on the internet*.”

### Social class

Social class, reflected in educational attainment and occupational status of both women and their husbands, significantly influenced access to reproductive health services. Low literacy levels were common, particularly among undocumented women. Participant 2 explained the need for better health education: “*In order for us to become familiar with the disease, it would be much better if doctors could give lectures or if there were videos available on the internet*.”

Most participants were housewives, while those who worked had long and irregular working hours that made attending health centers difficult. Participant 1 stated: “*My job is such that I am at work in the morning, and I can't come to the health center.*” Husbands’ low education level was also notable. In the undocumented group, 55% of husbands had no formal education. This lack of spousal education was linked to reduced support for women’s healthcare needs and lower household health literacy.

## 2. Intermediary determinants of health

### Living conditions

Poor housing conditions, shared accommodation, inadequate nutrition, and financial strain from unstable jobs restricted women’s ability to seek care. Participant 18 described: “*The house is very crowded, there are many tenants, and the pond water is dirty... the children get sick*.”

Participant 19 added: *“We have trouble buying food; we can't buy everything... if I had to spend money on a doctor, I would spend it on the kids*.”

### Bio-behavioral factors

Bio-behavioral factors, which include participants’ medical history and their perceived need for reproductive health services, significantly influenced health-seeking behaviors. Many women delayed or avoided care due to fear of deportation, financial constraints, and a low perceived urgency of preventive services. Some attributed physical symptoms to post-migration stress rather than seeking professional help. Participant 10, who had a long history of tuberculosis, explained how financial difficulties led her to self-treat gynecological issues: “*Due to financial constraints, she resorts to self-treatment at home for gynecological conditions and related issues.*” Participant 8 described a miscarriage during the journey to Iran: “*During the journey into Iran, I experienced a miscarriage accompanied by bleeding. The conditions were very harsh; we had no choice but to flee, and there was no time to treat my bleeding. I continued on in that state*.” Participant 19 highlighted how economic priorities affected self-care: “*We were more concerned about work, food, and shelter; we didn't have money for a doctor, and if I had to spend money on a doctor, I would spend it on the kids* These accounts demonstrate how previous health conditions, perceived need, and competing survival demands shaped women’s decisions regarding reproductive health care.

### Psychological factors

Migration-related stress, fear of arrest, living secretly, and fear of spousal abandonment were commonly reported. Participant 20 said: “*Every day they say they have to kick out Afghans from Iran... we always have the stress of being abandoned*.” Participant 8 described how spousal violence restricted her movement: “*When he's angry, he doesn't let me go outside. If I say I want to see a doctor, he tells me he'll take me later*.”

### Accessibility and health service delivery

The high cost of health services was repeatedly mentioned as a major financial-access obstacle. A member of the Midwifery Association stated: “*International organizations should provide financial resources, and the Ministry of Foreign Affairs, Ministry of Welfare, and Ministry of Health must work together to attract resources.*”

The lack of specialist doctors and language barriers due to untrained personnel hinder migrants’ access to services. Participant 15 said, “*I don't fully understand the language because my sister has to come with me”.* Additionally, the lack of integrated services discourages immigrants from seeking care. Participant 20 shared, “*My mother is sick and I constantly think about her. It was supposed to be better here, but it didn't happen. Every appointment with the doctor is in a different part of the city. I wish everything was in one hospital. We have to pay a lot for transportation, and we don't even know where it is*.”

Access to equal treatment facilities through mobile clinics was emphasized by the head of the NGO: “*These women do not go anywhere to report their pregnancies because they fear that their illegal status will cause them to be expelled from Tehran or the big city. They do not consider themselves to have any rights.*” Mobile clinics were suggested as an effective approach for providing urgent primary health services like sexual health education and care.

A service provider and NGO manager emphasized the strategic importance of mobile units: “*Mobile clinics are more than just a medical service; they are a lifeline for women who live in constant fear of leaving their immediate surroundings. By bringing reproductive screenings and basic care directly to their doorstep, we remove the barriers of distance and the paralyzing fear of being arrested on the way to a formal hospital.*”

### Quality of services

Participants emphasized the need for dignity-centered services and adequate consultation time. Participant 10 explained, “*We don't go to the doctor often, but I understand the language. However, here they give medicine for everything and don't fully listen to the patient's concerns*.” She also suggested that services for migrants should be provided by people from their own community, saying, “*We need to train these individuals and establish service centers specifically for them, which can provide better assistance and education.*” Additionally, she recommended intermediaries like those in health center programs to educate and support migrants, with services either free or supported by NGOs.

## 3. Cross-cutting Factors

### Social cohesion and social capital

This category refers to the quality of relationships between migrants and the host society, focusing on trust in institutions and social acceptance. It acts as a psychological gateway to healthcare. NGO official noted: *“Migrants often fear participating... they are not even willing to come inside the center due to the negative perceptions Iranians may have towards them.”*

### Social support networks

This category refers to the functional assistance (emotional, financial, and informational) provided by immediate circles (family, peers and NGOs) to compensate for systemic gaps. Participant 19: *“We consult one another in our female circles... a friend took me to a traditional midwife... we have no one but ourselves.”* Participant 3: *“We rely on relatives and fellow townspeople. If I have an illness, I ask them where to go.”*

## Discussion

By Using the World Health Organization’s Social Determinants of Health (WHO-SDH) framework, this study explores the complex realities of reproductive health access among Afghan migrant women in Iran. A key finding is the intersectional nature of the barriers these women face. Factors such as financial instability and the weakness of formal social support do not operate in isolation; rather, they intersect with legal status, gender norms, and cultural expectations. This intersectional dynamic acts as a powerful underlying force that shapes women’s health-seeking behavior, intensifies psychological stress, and connects broader structural policies with their daily lived experiences.

This intersectional dynamic can be conceptualized as a triad of vulnerability, in which being a woman, being an (often undocumented) migrant, and living within a patriarchal social structure combine to compound disadvantage. Each dimension alone constrains access to reproductive health services; together they interact multiplicatively rather than additively, so that legal precarity heightens the effect of patriarchal restrictions on women’s agency, which in turn deepens the isolation produced by undocumented status. The findings reported under Governance and Legal Status and Cultural and Social Values (Table 3) illustrate how this triad manifests in practice, from reliance on male relatives to access basic services to the compounded fear of both deportation and social sanction.

## 1. Structural determinants of health

### 1.1. Governance and legal status

Our findings reveal that restrictive migration policies and precarious legal status function as fundamental structural barriers, severely obstructing access to sexual and reproductive health (SRH) services. These obstacles extend into the broader civic sphere—including restrictions on property ownership, banking, and even SIM card registration—effectively deepening the marginalization of Afghan migrants. Consistent with prior qualitative work conducted among Afghan refugees within Iran's own health service delivery system, these legal frameworks drive economic inequality, social isolation, and identity crises [20], as the rights and services available are strictly contingent upon residency status [21]. Our findings extend this literature by situating it within Phelan and colleagues’ fundamental-cause framework [22], originally developed from the US sociological literature on health inequalities rather than from migrant or Iranian settings; applying this theoretical lens to our Iran-specific qualitative data suggests that legal status functions less as a fixed background variable, as the original theory implies, and more as an actively enforced mechanism of exclusion.Crucially, the persistent fear of arrest and deportation acts as a potent psycho-structural deterrent, forcing many undocumented individuals to avoid essential healthcare altogether to remain invisible to authorities [22].

### 1.2. Macroeconomic policy

Our findings illustrate that livelihood instability and legal employment restrictions force migrant families to prioritize immediate survival over vital healthcare. This financial deadlock, compounded by the lack of insurance, leads to Catastrophic Health Expenditures (CHE) and high out-of-pocket payments, triggering a downward spiral into absolute poverty, food insecurity, and mental health deterioration [23]. Furthermore, relentless financial strain appears to exacerbate the risk of domestic violence within the household. This pattern is consistent with a systematic review of migrants’ health in Iran [23], although that review synthesized largely quantitative and administrative data rather than the first-person qualitative accounts our design captures, meaning our findings add explanatory depth—the lived mechanisms linking financial strain to household violence—that a review-level synthesis cannot provide. Eliminating financial barriers to reproductive services not only reduces inequity but also serves as a long-term cost-saving strategy by preventing high-risk deliveries and the spread of communicable diseases [24].

### 1.3. Cultural and social values

Cultural values and religious traditions exert a dual influence on reproductive health outcomes. We found that a persistent preference for home births—deeply rooted in traditional practices—often results in severe complications when medical infrastructure is absent [25]. These challenges are compounded by cultural and linguistic disparities that, during complex clinical encounters, tend to erode mutual trust and heighten patient distress [26,27]. Furthermore, rigid gender norms and traditional roles continue to silence discussions around menstrual health and sexually transmitted infections (STIs), creating significant barriers to disclosure [28,29]. For many participants, healthcare is only perceived as legitimate when it aligns with their religious principles [16]. While adapting services to respect these religious frameworks can improve uptake, we argue that true informed decision-making remains dependent on broader efforts to enhance digital literacy and provide professional, culturally-attuned counseling [30].

### 1.4. Social policy

Social policies concerning migrants are inherently tied to biosecurity and the fundamental entitlement to welfare. Our results suggest that systemic gaps, particularly unequal access to social safety nets and health insurance, leave migrants acutely vulnerable to catastrophic health expenditures. Moreover, policies governing housing and urban marginalization serve as critical health determinants, directly dictating living conditions and environmental sanitation. This is directly corroborated by two Iran-based, community-level quantitative studies of Afghan women that similarly found excluding migrants from formal insurance frameworks does more than restrict prenatal care; it actively drives food insecurity and adverse pregnancy outcomes [31,32]. Because these studies used structured surveys, they can quantify the scale of the problem in ways our qualitative design cannot, while our interviews in turn surface the underlying decision-making processes and lived trade-offs behind their statistical associations—the two approaches are complementary rather than contradictory. Consequently, we argue that reforming social policies to ensure minimum welfare standards and Universal Health Coverage (UHC) is a strategic necessity to mitigate profound biological and social inequalities [33].

### 1.5. Public policy

Our findings reveal that while public policy in this domain targets macro-management and international cooperation, a persistent lack of coordination between government bodies, international agencies, and NGOs continues to undermine the sustainability of migrant reproductive health services. Public policies centered on media and awareness-building are not just communicative tools; they are decisive in dismantling stigma and fostering social integration [34].In the digital era, policymaking must also pivot toward infrastructure; currently, limited access to online resources and health platforms has entrenched a new layer of ‘information poverty’ among migrants echoing a comparative Spain–Sweden study of immigrants’ digital health literacy [35]. Both of these sources describe European policy environments with functioning national health-insurance and digital-infrastructure baselines; Iran's context, additionally shaped by international economic sanctions and a large undocumented population outside any insurance scheme, presents a materially different starting point, which is likely why our data extend this literature by showing that the central policy gap is not merely one of awareness, but of the structural policy instruments themselves.

### 1.6. Social class

Applying an intersectional and gender lens reveals that structural determinants impact Afghan migrant women unevenly, with social class—specifically the husband’s education—acting as a pivotal driver of reproductive health choices. Our findings confirm that health literacy is fundamentally linked to informed decision-making and superior outcomes [36]. That cohort study was conducted in a general clinical population rather than among migrants specifically, so its relevance to our findings is at the level of the general health-literacy mechanism rather than a migrant-specific comparison. Lower paternal education often reinforces patriarchal norms, curtailing female autonomy and making it harder for women to negotiate access to contraception or prenatal care. This vulnerability is compounded when intersected with legal precarity and economic hardship [37]. Because that study measured IPV prevalence numerically across a defined sample, while our data are drawn from in-depth narrative accounts, our contribution is a more specific, process-level finding: undocumented women with low educational backgrounds face a ‘triple burden’: restricted agency, low household health literacy, and a paralyzing fear of deportation. This explains the disproportionately poorer outcomes observed in these specific subgroups [31,37]. Furthermore, while there is a strong demand for digital health education, limited access to technology creates a new dimension of ‘information poverty’ and digital inequality among this population [38].

## 2. Intermediary determinants of health

### 2.1. Living conditions

Our results highlight a stark reality: Afghan migrant women are often forced to prioritize ‘basic survival’ over self-care and preventive services due to their precarious livelihood conditions. When faced with inadequate material circumstances, including substandard housing, the lack of privacy in shared accommodations, chronic malnutrition, and the relentless financial strain of unstable employment, there is simply little cognitive or physical room left to focus on reproductive health. This finding is consistent with two Iran-based community surveys of Afghan women [31,32], which similarly confirm that adverse pregnancy outcomes among this population are not accidental, but are closely tied to systemic failures such as food insecurity and the absence of health insurance; as above, their survey design quantifies the association while our qualitative accounts illustrate the everyday reasoning behind it. With only approximately one-third of these women receiving what is considered standard care, it is clear that for the majority, prenatal health remains a luxury they cannot afford amidst the struggle for daily survival [31,32].

### 2.2. Psychosocial factors

We found that migration-related stressors, ranging from chronic livelihood insecurity and the looming fear of deportation to the emotional toll of family separation—exert a profound and negative pressure on the mental health of Afghan women. These persistent stressors are not merely psychological burdens; they are closely tied to elevated levels of anxiety and depression, which we observed to have a direct, detrimental impact on their sexual and reproductive health outcomes [39]. This corroborates a European call-to-action on mental health among migrant and refugee pregnant women [39], though that source is a position paper synthesizing the case for intervention rather than an empirical study, and it addresses pregnant migrants broadly rather than the Afghan-Iranian population specifically. Our data suggest that women enduring long-term psychological distress are significantly more prone to clinical disorders and often show a marked reluctance to engage with medical services [40].

Perhaps the most sensitive finding of this study is how psychosocial stressors intersect with physical health through the lens of Intimate Partner Violence (IPV). The precarious nature of migration appears to exacerbate the risk of such violence within the household. This is particularly concerning given that IPV is known to increase vulnerability to reproductive tract infections and STIs [37]. Beyond the immediate psychological trauma, domestic violence strips women of their autonomy, creating a pervasive climate of fear that makes seeking timely medical help nearly impossible. These challenges are further complicated by rigid gender norms; many women in our study remained dependent on their husband’s permission or accompaniment to visit health centers. This practice is deeply embedded in patriarchal structures that fundamentally restrict their mobility and independent decision-making power.

### 2.3. Bio-behavioral factors

At the bio-behavioral level, we found that health-seeking actions are primarily dictated by the participants’ medical history and their own ‘perceived need.’ Driven by a deep-seated fear of legal repercussions and a pervasive mistrust of institutions, many women adopt avoidance as a core coping mechanism—a strategy that inevitably leads to the dangerous postponement of essential care [25]. This dynamic parallels a Tehran-based study of the determinants of reproductive health behaviors among Afghan immigrant women [41], which used a sociological survey design rather than in-depth interviews; the convergence of a survey-based and an interview-based study on the same underlying pattern strengthens confidence that the association is not an artifact of either method. This delay is further compounded by a low valuation of preventive services, such as screenings, which are frequently sidelined in the face of immediate economic precarity. Our data also reinforce the link between limited educational attainment—among both the women and their spouses—and suboptimal self-care practices [25]. It is crucial to recognize that the health literacy and overall status of this migrant population are not isolated issues; they directly influence the social stability and public health of the host society at large [41]. In several instances, participants viewed their physical pain merely as a somatic manifestation of post-migration stress, failing to see the necessity for clinical screening. Most distressingly, the cumulative trauma of the migratory journey in our study was linked to severe physical complications, including the tragic occurrence of miscarriages.

### 2.4. Health system accessibility and service quality

From a health system standpoint, ingrained physical and structural obstacles, ranging from the geographic distance of health centers to restrictive operating hours and a lack of integrated care, directly impede how these women utilize services, a finding that echoes earlier reports [20]. Our data further reveal that the cultural and linguistic mismatch between providers and the migrant community significantly worsens this gap [23]. Both of these sources, like our own study, are grounded in the Iranian context, which strengthens the comparability of the structural picture they describe; where they diverge from our findings is chiefly in scope, since [20] and [23] describe system-level service gaps generally, whereas our data specify how those gaps are experienced and navigated by women seeking reproductive care in particular. When consultation times are rushed and communication lacks cultural sensitivity, it often breeds a sense of ‘clinical alienation’ and stigma. We found that these discriminatory undertones do more than just discourage visits; they actively prevent women from disclosing sensitive reproductive concerns or sticking to necessary follow-up protocols [31].To bypass these hurdles, the women in our study voiced a clear preference for digital alternatives, such as virtual platforms and short videos produced in their native language. Moreover, they pointed to mobile clinics as a vital, pragmatic solution for accessing both urgent care and reproductive education, especially for those whose circumstances make visiting a fixed healthcare facility nearly impossible. Beyond service organization, the high cost of care itself remains a central financial-access barrier: eliminating out-of-pocket costs for reproductive services would not only reduce inequity but also serve as a long-term cost-saving strategy by preventing high-risk deliveries and the spread of communicable diseases [24].

## 3. Cross-cutting factors

### 3.1. Social support networks: Formal and informal

Where formal state support falls short, informal networks, spanning family ties, friendships, and non-governmental organizations (NGOs), step in as essential compensatory mechanisms. Our data indicate that for many Afghan women, navigating the complexities of the healthcare system is a journey guided primarily by peers and fellow citizens. This reliance on informal channels aligns with recent evidence suggesting that such networks do more than just provide emotional buffering; they actively enhance health literacy and serve as a vital link to reproductive services, particularly in mitigating mental health risks as suggested in the European literature on migrant maternal mental health [39]. As noted above, that source is a position paper rather than a primary study of social-network effects, so we treat this as a broadly consistent conceptual parallel rather than a direct empirical replication. The potential of the formal arm of this support, the NGOs, remains largely stifled. These organizations grapple with severe budgetary constraints and legal hurdles that limit their capacity to offer the comprehensive care required. We argue that bolstering financial and legal support for these NGOs is not just a policy recommendation, but a direct pathway to improving the quality and reach of reproductive health services for this marginalized population [42].

### 3.2. Social cohesion and social capital

Our findings reveal that social capital and cohesion are not merely static backgrounds, but active, dual-edged forces that dictate how reproductive health is accessed. We observed that Iranian migration policies and legal frameworks have inadvertently fostered a sense of social isolation and economic disparity. These structural pressures create significant identity hurdles for Afghan migrants, effectively bottlenecking their integration into the broader support systems of the host society.

As a result, strengthening intra-group (bonding) social capital becomes a survival strategy rather than a choice. In line with a systematic review of qualitative studies on social capital and SRHR in humanitarian settings more broadly [43]—which was not specific to Afghans or to Iran but synthesized diverse displacement contexts, the social resources available to these women are almost exclusively of the ‘bonding’ variety, with ‘bridging’ social capital—those vital links to individuals and resources outside their ethnic circle, remaining largely out of reach [43]. The consistency between a multi-setting humanitarian review and our single-city Iranian sample suggests this bonding/bridging imbalance may be a broadly generalizable feature of displacement rather than one specific to the Iranian policy environment.These internal network mechanisms, including informal social control and collective action, function as a double-edged sword; they can either smooth the path to healthcare or act as a barrier, depending on the specific cultural norms dominant within the group. Ultimately, this deficit in inter-group cohesion does more than just isolate the community; it actively erodes trust in public institutions and reinforces a systemic avoidance of formal healthcare facilities.

Despite persistent structural barriers, Iran’s Primary Health Care (PHC) system remains a key institutional facilitator. By integrating all migrants—regardless of their legal status—into the national electronic health record (SIB), the system technically ensures that maternal care and vaccinations are free of charge. This reflects a formal commitment to the health rights of the migrant population. However, we found that actual reproductive health outcomes for Afghan women in Tehran are not determined by service availability alone; instead, they emerge from a complex interplay between these services and individual bio-behavioral factors. Our data, aligning with the Canadian literature on immigrant and refugee women's mental health and well-being discussed above [40], show that inadequate self-care—particularly regarding psychosocial and sexual health—remains a major challenge. That the same pattern recurs in both a North American immigrant population and our Iranian migrant sample suggests self-care avoidance under psychosocial strain may be a cross-context feature of migration itself, rather than one produced solely by Iran's specific policy environment. These behaviors are deeply rooted in socio-demographic realities. For instance, we observed that when both women and their husbands have higher educational attainment, there is a clear positive correlation with effective PHC utilization. In contrast, women with limited education often struggle to navigate the system or prioritize preventive care. This suggests that while the institutional ‘door’ to access is open, socio-economic conditions and individual health literacy remain the real gatekeepers of health utilization.

These findings do more than just highlight challenges; they point toward a critical need for interventions that go beyond the clinic walls. Addressing issues like Intimate Partner Violence (IPV) and improving social support networks are just as vital as providing medical care. Effectively tackling these multifaceted problems will require more than local action—it demands a serious, collaborative effort between government bodies and international agencies.

### Implications for policy and practice

These findings carry several concrete implications for policy and practice. First, because legal precarity was the determinant most consistently reported as amplifying every other barrier, extending basic reproductive health entitlements independent of documentation status would likely yield the largest equity gain per policy investment. Second, the “register-and-wait” pattern observed in public-policy implementation indicates that expanding formal enrollment mechanisms alone is insufficient; policies must be paired with functional referral pathways and subsidized insurance to translate entitlement into actual care. Third, given the centrality of information poverty across both the public-policy and bio-behavioral domains, investment in multilingual digital health platforms and community-based peer education may offer a comparatively low-cost, high-reach intervention. Finally, integrating routine screening for intimate partner violence into existing migrant-serving primary care contacts could address a risk factor that our findings suggest is currently invisible to the formal health system. Collectively, these priorities point toward interventions operating simultaneously at the structural (legal and policy) and intermediary (service-delivery and information) levels, consistent with the intersectional dynamic described above.

### Strengths and limitations

Regarding limitations, our sampling was focused on Afghan women in Tehran, which may limit how much we can generalize these findings to other migrant populations or regions. Furthermore, since we relied heavily on qualitative interviews, the results are naturally shaped by the personal interpretations and lived experiences of the participants. For future research, we suggest expanding this inquiry to other Iranian cities and diverse migrant groups. Such comparative work is essential to build a more comprehensive and generalizable model of migrant health in the region.

Although ethnic background and religious affiliation were recorded for all participants, with the majority identifying as Hazara/Shia (n = 17) and a minority as Pashtun/Sunni (n = 3), the sample size was insufficient to draw meaningful comparisons across ethnic or religious groups. This is a notable limitation, as Afghanistan's diverse ethnic communities (Hazara, Pashtun, Tajik, Uzbek) carry distinct historical experiences of persecution, cultural norms around gender and healthcare, and varying levels of trust in institutions. These factors are likely to shape reproductive health-seeking behavior differently across groups. Future research should employ purposive sampling strategies that ensure adequate representation of each ethnic community to enable rigorous comparative analysis.

## Conclusion

Using the WHO Social Determinants of Health framework, this study examined structural, intermediary, and cross-cutting barriers to reproductive health access among Afghan migrant women in Iran. Despite Iran's robust primary healthcare infrastructure and the technical integration of migrants into the national electronic health record (SIB), actual access remains suboptimal. Legal invisibility, economic hardship, and patriarchal norms intersect to trap women in a restrictive cycle: fear of deportation, financial strain, and traditional gender roles push many to delay or avoid essential care, often resulting in dangerous coping mechanisms such as self-treatment, high-risk home births, or preventable miscarriages. Leaving this population outside the formal healthcare safety net is both a human rights concern and a strategic public health risk, raising emergency care costs and the potential for communicable disease spread.

Addressing this requires structural change rather than mere service expansion: decoupling healthcare access from residency status, providing culturally tailored care, involving men in reproductive health programs, and strengthening mobile clinics for hard-to-reach groups. Investing in the reproductive health of Afghan migrant women is ultimately an investment in Iran's broader health security and social stability. As the qualitative phase of a sequential exploratory mixed-methods program, this study's findings have informed a quantitative instrument tested through structural equation modeling and will guide a forthcoming policy package. Future research should prioritize ethnic diversity within Afghan migrant samples (Hazara, Tajik, Pashtun, Uzbek) and examine variation by age, migration pathway, and pre-migration reproductive health knowledge to strengthen generalizability.

## Supporting information

S1 Checklist Consolidated Criteria for Reporting Qualitative Research (COREQ) checklist. (DOCX)

S1 Text The semi-structured interview guides used for Afghan migrant women and key informants. (DOCX)
