# Supplementary material for: Social determinants of access to reproductive health services among Afghan migrant women in Iran: A qualitative study
Source: PLOS Glob Public Health. 2026 Oct 15;6(10):e0007406. doi: 10.1371/journal.pgph.0007406 (PMC13671505; doi:10.1371/journal.pgph.0007406)
Supplement: Supplementary file 1 [file pgph.0007406.s001.docx]

Consolidated criteria for reporting qualitative studies (COREQ): 32-item checklist

Social Determinants of Access to Reproductive Health Services among Afghan Migrant Women in Iran: A Qualitative Study

| No. Item | Guide questions/description | Reported on Page # |
| --- | --- | --- |
| Domain 1: Research team and reﬂexivity |  |  |
| *Personal Characteristics* |  |  |
| 1. Inter viewer/facilitator | Which author/s conducted the inter view or focus group? | The semi-structured in-depth interviews were primarily conducted by Zahra Bayat Jozani. An Afghan midwifery bachelor's student from Tehran University of Medical Sciences provided assistance with translation, cultural interpretation, and verification of linguistic and contextual accuracy during some interviews when required. |
| 2. Credentials | What were the researcher’s credentials? E.g. PhD, MD | PhD student |
| 3. Occupation | What was their occupation at the time of the study? | I have 23 years of practical and research experience working with vulnerable women, particularly in the areas of HIV/AIDS prevention and care, and support for sex workers. This background has provided me with in-depth insight into the sexual and reproductive health challenges faced by these populations, through fieldwork, counseling, training, and the design of harm-reduction and intervention programs in related health and support centers.  Currently, as a clinical instructor at the School of Nursing and Midwifery, Tehran University of Medical Sciences, I am supervising and guiding a doctoral thesis on 'Access to Reproductive Health Services among Migrants.' This qualitative research, guided by the Social Determinants of Health (SDH) framework, examines structural and intermediary barriers to reproductive health service access for migrant women (particularly Afghan women) in Iran.  This long-term combination of clinical-social experience, teaching, and doctoral-level research supervision enables a comprehensive and practice-oriented perspective on the reproductive health issues of marginalized groups |
| 4. Gender | Was the researcher male or female? | Female |
| 5. Experience and training | What experience or training did the researcher have? | Zahra Bayat Jozanihas completed formal training courses in qualitative interviewing techniques, qualitative data analysis, and all relevant academic writing and research methodology modules as part of the doctoral program at Tehran University of Medical Sciences.  Dr. Zohreh Mahmoodi, Associate Professor and Director of the Social Determinants of Health (SDH) Research Center, served as the methodological consultant and was actively present throughout the coding process to ensure rigor and alignment with the directed content analysis approach. All coding decisions, categories, and themes were systematically reviewed and validated with the principal supervisor, Dr. Seyedeh Tahereh Mirmolaei, Professor and main thesis advisor.  This structured supervision and training ensured methodological trustworthiness, credibility, and confirmability of the qualitative findings. |
| *Relationship with participants* |  |  |
| 6. Relationship established | Was a relationship established prior to study commencement? | Yes, a relationship was established prior to study commencement to build trust and facilitate participation, particularly among Afghan migrant women.  An Afghan peer (a midwifery student at Tehran University of Medical Sciences) was involved as a facilitator. This peer helped introduce the study to potential participants from the Afghan community, leveraging shared language (Dari/Persian) and cultural background to reduce suspicion and enhance comfort.  The main researcher (Zahra Bayat Jozani) is well-known in relevant community and professional circles due to many years of experience in training and providing services to vulnerable women, including migrants. She has previously conducted educational classes for migrants in collaboration with the municipality (Tehran Municipality women’s centers), during which she introduced herself as a clinical instructor and researcher from Tehran University of Medical Sciences. In those sessions and during recruitment for this study, she clearly explained that the research was purely academic, had no therapeutic or clinical intervention component, and would not affect participants’ healthcare access or legal status in any way.  This pre-existing familiarity, combined with transparent communication and the involvement of a trusted Afghan peer, helped establish rapport, address concerns (especially about confidentiality and deportation risks), and encourage open participation in the interviews. No prior clinical or personal therapeutic relationship existed with any participant; all interactions were strictly for research purposes. |
| 7. Participant knowledge of the interviewer | What did the participants know about the researcher? e.g. personal goals, reasons for doing the research | Participants were clearly told that the study was part of her doctoral thesis at Tehran University of Medical Sciences, and the main reason for conducting the research was to better understand and document the barriers Afghan migrant women face in accessing reproductive health services in Iran. The goal was explained as academic and policy-oriented: to generate evidence that could help improve health services and reduce inequalities for migrant women in the host country. |
| 8. Interviewer characteristics | What characteristics were reported about the inter viewer/facilitator? e.g. Bias, assumptions, reasons and interests in the research topic | The main interviewer was Zahra Bayat Jozani main researcher of the study. The following characteristics were relevant and are reported here for transparency and reflexivity:   - Professional background and experience: She has 23 years of clinical, educational, and research experience working with vulnerable women, including migrant populations, women living with HIV/AIDS, and sex workers. Her work has focused on sexual and reproductive health, harm reduction programs, counseling, training, and community-based interventions in Tehran. This extensive experience has shaped her strong interest in equity in health access for marginalized groups, particularly migrant women. - Current role: Clinical instructor at the School of Nursing and Midwifery, Tehran University of Medical Sciences, where she supervises doctoral theses, including this PhD project on access to reproductive health services among migrants. - Reasons and interests in the research topic: The researcher has a long-standing professional and academic interest in social determinants of health, health inequities faced by migrant and refugee women, and improving access to culturally sensitive reproductive healthcare in Iran. This study forms part of her doctoral thesis and is driven by the recognition that Afghan migrant women represent a highly vulnerable group with documented barriers to reproductive health services in the Iranian context. Her motivation is academic (contributing to evidence-based policy and practice) and ethical (advocating for health equity and reducing disparities for underserved populations). - Potential biases and assumptions:   - Positive bias: Due to her extensive work with vulnerable women, she may have a pre-existing empathy and advocacy orientation toward improving service access for migrants.   - Assumption: She assumed that structural factors (e.g., legal status, financial barriers, discrimination) play a major role in limiting access, based on prior literature and her professional experience.   - To mitigate these potential biases, the study used directed content analysis guided by the WHO SDH framework, involved independent supervision by Dr. Zohreh Mahmoudi (methodological consultant and SDH Research Center Director) throughout coding, and had all coding decisions and themes systematically reviewed and validated by the principal supervisor, Dr. Seyedeh Tahereh Mirmolaei. Member checking with participants and peer debriefing were also employed to enhance confirmability and reduce researcher bias influence on findings. |

| Domain 2: study design |  |  |
| --- | --- | --- |
| *Theoretical framework* |  |  |
| 9. Methodological orientation and Theory | What methodological orientation was stated to underpin the study? e.g. grounded theory, discourse analysis, ethnography, phenomenology, content analysis | The methodological orientation underpinning this study was directed content analysis (also known as deductive content analysis).  This approach was explicitly chosen and stated in the study protocol and manuscript as follows:   - Directed content analysis was employed, guided by the World Health Organization's Social Determinants of Health (SDH) conceptual framework as the initial deductive coding structure. - The SDH framework provided the predefined categories (structural and intermediary determinants) against which the data were analyzed. - While the analysis remained primarily deductive (theory-driven), emergent (inductive) codes and subcategories were allowed to arise from the data to refine, expand, or challenge the initial framework, ensuring both structured and flexible interpretation of participants' experiences.   This orientation was selected because it allowed for systematic examination of the research question (barriers to reproductive health service access among Afghan migrant women in Iran) within an established theoretical model (WHO SDH), while still permitting the emergence of context-specific insights from the participants' narratives. |
| *Participant selection* |  |  |
| 10. Sampling | How were participants selected? e.g. purposive, convenience, consecutive, snowball | Participants were selected using purposive sampling with a maximum variation strategy to ensure a diverse and information-rich sample.  Detailed selection process:   - Afghan migrant women participants (n=20): Purposive sampling was employed to deliberately select women of reproductive age (18–45 years) who had resided in Iran for at least one year. Maximum variation was applied to capture a wide range of experiences across key characteristics: residency status (documented/undocumented, Amayesh cardholders, passport holders, Iranian ID through marriage), socioeconomic background, duration of stay in Iran, educational level, parity, and prior experiences with reproductive health services. Recruitment occurred at public health centers affiliated with Tehran University of Medical Sciences and municipality-supported women’s educational and recreational centers in Tehran. An Afghan midwifery student peer facilitator assisted in identifying and approaching potential participants from the Afghan community to enhance trust and access. - Key informants (n=10): Purposive sampling was used to select healthcare providers and experts with relevant professional experience in reproductive health, midwifery, migrant health services, or related policy/NGO work. Diversity was sought across settings (public health centers, hospitals, midwifery associations, NGOs, private counseling centers) and roles (clinicians, policymakers, educators, counselors). Initial contacts were made through professional networks and gatekeepers in Tehran’s reproductive health community.   No convenience, consecutive, or snowball sampling was used. Sampling continued until theoretical saturation was reached (no new themes or insights emerged from additional interviews), which was determined through ongoing analysis and discussion with supervisors.  This purposive, maximum-variation approach was explicitly chosen to generate rich, in-depth data on the barriers to reproductive health service access among Afghan migrant women in Iran, as aligned with the qualitative nature of the study and the WHO Social Determinants of Health framework. |
| 11. Method of approach | How were participants approached? e.g. face-to-face, telephone, mail, email | Participants were primarily approached face-to-face to establish trust, explain the study clearly, address concerns, and obtain informed consent in person, given the sensitive nature of the topic (reproductive health barriers among Afghan migrant women) and the need to reduce suspicion, especially among undocumented individuals.  Detailed approach process:   - Face-to-face recruitment: The main researcher (Zahra Bayat Jozani), sometimes accompanied by the Afghan midwifery student peer facilitator, approached potential participants directly at the recruitment sites: public health centers affiliated with Tehran University of Medical Sciences and municipality-supported women’s educational/recreational centers in Tehran. Initial contact involved a brief, respectful introduction of the researcher, explanation of the study purpose (in Persian/Dari), assurance of confidentiality and voluntary participation, and distribution of the participant information sheet. This face-to-face method allowed for immediate clarification of questions and helped build rapport. - Telephone verification/follow-up: After initial face-to-face contact and expression of interest, the researcher confirmed eligibility, availability, and willingness to participate via telephone. Telephone calls were also used to schedule interviews, reconfirm consent, and conduct member checking (verification of key points or clarification of data) after interviews when needed. No recruitment was done solely via telephone; it served only for confirmation, scheduling, and follow-up. - No mail, email, or other indirect methods were used for initial approach, as personal, direct contact was essential to ensure understanding, address cultural/language concerns, and minimize potential fear or mistrust related to migrant status and research participation.   This combined face-to-face approach with telephone confirmation ensured ethical, culturally sensitive recruitment and high participant engagement. |
| 12. Sample size | How many participants were in the study? | 20 Afghan reproductive age women and 10 key information |
| 13. Non-participation | How many people refused to participate or dropped out? Reasons? |  A total of 3 Afghan migrant women declined to participate after initial face-to-face approach and explanation of the study during recruitment. Reasons given (verbally, in Persian/Dari):   - Fear of potential risks related to their undocumented or precarious legal status (concern that participation might draw attention or affect their residency situation). - Lack of time due to heavy household and childcare responsibilities. - General discomfort or reluctance to discuss sensitive personal topics (reproductive health experiences) with an outsider, even though confidentiality was assured.    No participants dropped out after providing informed consent and starting the interview process. All 20 Afghan migrant women who agreed to participate completed their interviews fully. Similarly, all 10 key informants completed their interviews without dropout.   No refusals or dropouts occurred among the key informants (healthcare providers and NGO staff), likely due to their professional familiarity with the researcher and the non-sensitive nature of their questions (system-level barriers rather than personal experiences). |
| *Setting* |  |  |
| 14. Setting of data collection | Where was the data collected? e.g. home, clinic, workplace | Data collection (semi-structured in-depth interviews) took place at locations chosen by the participants to ensure their comfort, privacy, and convenience, given the sensitive nature of the topic (reproductive health barriers among Afghan migrant women) and potential concerns related to legal status or stigma.  Specific locations:   - Most Afghan migrant women interviews (majority of the 20 participants): Conducted in private rooms at public health centers affiliated with Tehran University of Medical Sciences or in quiet, private spaces at municipality-supported women’s educational and recreational centers in Tehran. These venues were familiar to participants (as places where they sometimes accessed health services or attended classes), which helped reduce anxiety and build trust. - Some interviews: Conducted via telephone at the participant’s preferred time and location (e.g., their home or another private setting), when face-to-face meetings were logistically difficult due to childcare responsibilities, transportation issues, fear of attending public venues, or other personal constraints. Telephone interviews were audio-recorded with explicit consent and followed the same interview guide. - Key informant interviews (the 10 healthcare providers and NGO staff): Held face-to-face in private offices or consultation rooms at their workplaces (e.g., public health centers, hospitals, midwifery association offices, NGO facilities, or private midwifery counseling centers in Tehran). These locations were convenient for professional participants and ensured a quiet, confidential environment.   No interviews were conducted in participants’ homes (to avoid intrusion and maintain privacy boundaries) or in public/open spaces. |
| 15. Presence of non-participants | Was anyone else present besides the participants and researchers? | Yes, in addition to the participants and the main researcher (Zahra Bayat Jozani), an Afghan midwifery student (bachelor's level, from Tehran University of Medical Sciences) was present as a peer facilitator during recruitment and some of the interviews.  Details:   - The Afghan midwifery student accompanied the main researcher at recruitment sites (public health centers and municipality-supported women’s centers) and during several face-to-face interviews with Afghan migrant women. She assisted with translation (Dari/Persian), cultural interpretation, and building immediate trust by speaking in the participants' native language and sharing a similar cultural background. - In cases where additional translation or clarification was needed (e.g., during initial approach, consent process, or interviews), participants were encouraged to involve their own acquaintances or family members (e.g., relatives or friends who spoke Persian/Dari fluently) if they preferred. These individuals were occasionally present to help with language support or provide emotional comfort, but only with the participant's explicit consent and at their request. They were not involved in the interview content, questioning, or data collection process itself. - No other individuals (e.g., healthcare staff, NGO workers, or unrelated third parties) were present during the interviews unless specifically requested by the participant for support.   This arrangement was designed to reduce language barriers, enhance cultural sensitivity, minimize power imbalances, and increase participant comfort and openness, especially for undocumented or newly arrived Afghan women. All non-research individuals were asked to leave before the formal interview began if their presence was not required for translation, and confidentiality was strictly maintained. |
| 16. Description of sample | What are the important characteristics of the sample? e.g. demographic data, date | This qualitative study explored the lived experiences of 20 Afghan migrant women in Iran, complemented by the perspectives of 10 purposively selected key informants with expertise in reproductive health, midwifery, and services for migrant and vulnerable populations.  Among the Afghan women, ages ranged from 21 to 45 years (mean = 28.6 years). The majority had married young, with age at first marriage ranging from 12 to 25 years (mean = 17.7 years; lower in the undocumented group: 16.8 years). Residency status was mixed: nine women (45%) had documented (legal) migrant status, while 11 (55%) were undocumented. Duration of residence in Iran varied substantially, from 1.2 to 33 years (median = 4.5 years; mean ≈ 6.2 years). Educational levels were generally low, with a median of 6.0 years of formal schooling overall (median 6.0 years in both subgroups); seven women (35%) had no formal education (illiterate), predominantly in the undocumented group (6/11, 55%).  Husbands ranged in age from 25 to 60 years (mean = 36.4 years) and were mostly employed in low-skilled occupations (e.g., manual laborers, farmers, guards, or small traders). Among husbands with reported data, education averaged ≈ 8.2 years, with 8 (40%) having no formal education. The number of children per woman ranged from 0 to 11 (mean = 1.6; median = 0), indicating a mix of nulliparous and multiparous participants, with higher parity more common among undocumented women (median = 2).  Detailed individual characteristics of the 20 Afghan migrant women are presented in Table 1 in article |
| *Data collection* |  |  |
| 17. Interview guide | Were questions, prompts, guides provided by the authors? Was it pilot tested? | y es, the interview guide, including main questions, follow-up prompts, and exploratory probes, was developed by the authors in consultation with expert feedback sessions and was used consistently in all interviews.  Details:   - The semi-structured interview guide was designed by the main researcher (Zahra Bayat Jozani) in collaboration with the principal supervisor (Dr. Seyedeh Tahereh Mirmolaei). - Expert input was obtained through consultation sessions with specialists in reproductive health and qualitative research methodology to ensure relevance, clarity, and cultural appropriateness. - The guide was developed based on the following sources:   - The World Health Organization's Social Determinants of Health (SDH) conceptual framework.   - Two scoping reviews conducted by the first author, focusing on barriers to reproductive health service access among migrant women.   - A review of the literature related to the experiences of Afghan migrant women in Iran. - Content of the interview guide included:   - Open-ended main questions exploring participants’ experiences, perceptions, and barriers to reproductive health services (e.g., antenatal care, family planning, prevention of sexually transmitted infections, maternal health).   - Follow-up probes to explore structural determinants (e.g., financial, legal, policy-related) and intermediary determinants (e.g., psychosocial, cultural, behavioral) in greater depth.   - Questions were prepared in Persian, with flexibility for translation/explanation into Dari when needed, supported by the Afghan midwifery student peer facilitator. |
| 18. Repeat interviews | Were repeat inter views carried out? If yes, how many? | No, repeat interviews were not carried out. |
| 19. Audio/visual recording | Did the research use audio or visual recording to collect the data? | Yes, audio recording was used to collect the data.  Details:   - All semi-structured in-depth interviews (both with Afghan migrant women and key informants) were audio-recorded with the explicit written and verbal consent of each participant. - Audio recording was carried out using a secure digital voice recorder to capture the full content of the interviews accurately for verbatim transcription and subsequent analysis. - No visual recording (e.g., video) was used at any stage of data collection, to protect participant privacy and reduce potential discomfort, especially among undocumented migrant women concerned about confidentiality or legal risks. - After transcription, all audio files were permanently deleted following verification of the transcripts' accuracy and completeness by the research team (main researcher and Afghan midwifery student assistant). The deletion occurred under supervision of the research team to ensure no identifiable data remained. - Participants were informed prior to the interview that recording was for research purposes only, that they could request to stop the recording at any time, and that the files would be securely stored during analysis and destroyed afterward.   This procedure was approved by the ethics committee and aligned with principles of confidentiality, informed consent, and data protection. |
| 20. Field notes | Were ﬁeld notes made during and/or after the inter view or focus group? | Yes during and after all interview |
| 21. Duration | What was the duration of the inter views or focus group? | Interviews lasted approximately 45–60 minutes and were held at participant-preferred locations (e.g., midwifery service rooms or women’s centers) or via telephone when necessary to accommodate preferences and ensure comfort. |
| 22. Data saturation | Was data saturation discussed? | Yes, data saturation was explicitly discussed and achieved.  Details:  Theoretical saturation was reached after completing interviews with 20 Afghan migrant women and 10 key informants. Saturation was determined when no new themes, categories, or insights emerged from the last three consecutive interviews with migrant women and the final two key informant interviews. This was assessed through ongoing concurrent data collection and preliminary analysis, regular team discussions (with the main researcher, principal supervisor Dr. Seyedeh Tahereh Mirmolaei, and methodological consultant Dr. Zohreh Mahmoudi), and review of emerging codes against the WHO SDH framework.  The decision to stop sampling was made jointly by the research team in June 2025, confirming that the data were sufficiently rich and redundant to comprehensively address the research aim: explaining structural and intermediary barriers to reproductive health service access among Afghan migrant women in Iran. Saturation was documented in the research log and reflective memos, ensuring methodological rigor and alignment with qualitative standards. |
| 23. Transcripts returned | Were transcripts returned to participants for comment and/or correction? | Yes; transcripts were checked via telephone. |
| Domain 3: analysis and ﬁndings |  |  |
| *Data analysis* |  |  |
| 24. Number of data coders | How many data coders coded the data? | The data were independently coded by two researchers |
| 25. Description of the coding tree | Did authors provide a description of the coding tree? | “Yes. The coding tree was clearly described, including themes, categories, subcategories, and final codes (Table 3).” |
| 26. Derivation of themes | Were themes identiﬁed in advance or derived from the data? | “Themes were derived from the data and then organized according to the World Health Organization’s social determinants of health framework.” |
| 27. Software | What software, if applicable, was used to manage the data? | No qualitative data analysis software was used; data were managed and coded manually. |
| 28. Participant checking | Did participants provide feedback on the ﬁndings? | Yes. Participant feedback on the findings was obtained through telephone follow-up. |
| *Reporting* |  |  |
| 29. Quotations presented | Were participant quotations presented to illustrate the themes/ﬁndings? Was each quotation identiﬁed? e.g. participant number | Yes. Participant quotations were presented to illustrate the themes, and each quotation was identified by a participant number. |
| 30. Data and ﬁndings consistent | Was there consistency between the data presented and the ﬁndings? | Yes. The presented data adequately supported the reported findings. |
| 31. Clarity of major themes | Were major themes clearly presented in the ﬁndings? | Yes. The findings clearly presented the major themes |
| 32. Clarity of minor themes | Is there a description of diverse cases or discussion of minor themes? | Yes. The findings included discussion of diverse cases and minor themes, reflecting variations in participants’ experiences. |
