# Supplementary material for: Social determinants of access to reproductive health services among Afghan migrant women in Iran: A qualitative study
Source: PLOS Glob Public Health. 2026 Oct 15;6(10):e0007406. doi: 10.1371/journal.pgph.0007406 (PMC13671505; doi:10.1371/journal.pgph.0007406)
Supplement: Supplementary file 2 [file pgph.0007406.s002.docx]

**S1 Text: Semi-Structured Interview Guide**

**Part A: For Afghan Migrant Women**

I, Zahra Bayat, a PhD student in Reproductive Health at Tehran University of Medical Sciences, am the principal investigator of the project "Development and Testing of a Model for Social Determinants Affecting Access to Reproductive Health Services
and Providing a Policy Package for Afghan Migrant Women Attending Healthcare Centers of Tehran University of Medical Sciences.
This study is part of my doctoral dissertation, and my objective in these interviews is to explore the needs and concerns regarding access to reproductive health services among Afghan migrant women based on the World Health Organization model.
If at any point you do not understand my questions, my colleague, an Afghan midwifery student, will assist us. The interview will last approximately 45 minutes, and as a token of appreciation, you will receive a small gift.

The interviews will be anonymous, and they will be recorded. You may refuse to answer any question or withdraw at any time.
If possible, please state your name and introduce yourself.

## 1. Demographic Information

2. **Opening Questions:**
   - Could you please tell me about your journey to Iran and your current living situation?
   - How would you describe your overall health since moving to Tehran?
3. **Structural Determinants (Legal & Policy):**
   - How has your residency status (having/not having an Amayesh card) affected your decision to visit a doctor?
   - Have you ever felt concerned about legal issues or deportation when seeking reproductive health services?
4. **Intermediary Determinants (Access & Quality):**
   - When you need reproductive care (e.g., prenatal care, family planning), where do you go? Why?
   - How do you feel about the cost of services? Does the Integrated Health System (SIB) help you receive free care?
5. **Sociocultural & Power Relations:**
   - Do you feel respected by healthcare providers? Have you experienced any discrimination?
   - How does your family or husband influence your access to these services?
6. **Closing:**
   - What do you think is the biggest barrier for Afghan women like you in staying healthy?

**Part B: For Key Informants**

*This guide focuses on systemic and policy perspectives.*

## 1. Demographic Information

2. Is there a difference in the provision of reproductive health services between migrants and Iranian citizens? Do migrants have access to all levels of healthcare services?
3. What is the status of insurance coverage and costs for reproductive health services for migrants in Iran?
4. Are there policies or regulations in place for providing reproductive health services to migrants? Have these policies been developed based on needs assessments?
5. Are these programs evaluated, implemented, and supported effectively?
6. Are the objectives of reproductive health service programs for migrants clearly defined?
7. Do these programs incorporate behavior change models?
8. Are key stakeholders and NGOs involved in planning and implementing reproductive health programs for migrants?
