# Supplementary material for: Social determinants of access to reproductive health services among Afghan migrant women in Iran: A qualitative study
Source: PLOS Glob Public Health. 2026 Oct 15;6(10):e0007406. doi: 10.1371/journal.pgph.0007406 (PMC13671505; doi:10.1371/journal.pgph.0007406)
Supplement: Supplementary file 8 [file pgph.0007406.s008.docx]

**PLOS Global Public Health**

Social Determinants of Access to Reproductive Health Services among Afghan Migrant Women in Iran: A Qualitative Study **R3**

Reviewer comments

Dear Editor,

Thank you for your positive feedback and for sharing the final round of comments on our manuscript, "Social Determinants of Access to Reproductive Health Services among Afghan Migrant Women in Iran: A Qualitative Study." We appreciate the reviewer's careful reading and constructive suggestions.

We are pleased to confirm that all the comments raised have been addressed, and a revised version of the manuscript is enclosed. A point-by-point response to each comment is provided below for the reviewer's reference.

We thank the reviewer once again for their valuable input throughout the review process, which has substantially strengthened the manuscript.

Sincerely,
Zahra Bayat Jozani and co-authors

1. Line 45: delete extra full stop at the end of the sentence

Done. The extra full stop has been deleted.

2. Line 70: Insert space after the full stop “(6).According...”

Done. A space has been inserted: "(6). According..."

3. Line 72: Insert a numbered citation for the reference: “…Levesque et al…”

Done

4. Lines 75-77: Rewrite sentence to clarify what is meant by “officially”. Is it by policy decree or some national law, or by verbal commitment of national officials?

Done. The sentence has been revised to specify the legal basis: *"Although Iranian law — including constitutional provisions on the right to health and social security (Articles 29 and 43) and the mandatory insurance clause for foreign nationals under the Sixth Five-Year Development Plan (Article 70) — has been interpreted as establishing a legal commitment to provide primary healthcare, including maternal and child health services, to Afghans regardless of legal status, a significant gap persists between this legal entitlement and actual utilization*

5. Lines 116-117: You may use acronyms for WHO and SDH, earlier introduced in the manuscript rather than writing again in full and inserting acronym.

Done. Full terms have been replaced with the previously introduced acronyms.

6. Line 159: Separate the sub-heading and the sentence: Start the narrative on Trustworthiness on a new paragraph

Done.

7. Line 190: “Findings:..” Delete the colon after “Findings”

Done.

8. Line 371: “Discussion:” Delete colon after Discussion.

Done.

9. Lines 390-609: Would be better to not have too many sub-headings and to let the discussion flow. Throughout the discussion, draw out contrasts, besides the similarities from other studies, in their findings and methods, as well as settings. It is not always apparent in what settings the studies being cited originate (whether Afghanistan or elsewhere). Including the country contexts would help readers make informed comparisons. Also, critique the studies’ methods to enable readers judge the strength of evidence and the conclusions being compared with the present study.

We thank the reviewer for this comment. We would like to clarify that the sub-headings in the Discussion section were introduced in the previous round of revision at the explicit request of an earlier reviewer, who asked that the barriers be organized under headings corresponding to the structural, intermediary, and cross-cutting levels of the SDH framework. The original submitted version of the manuscript did not include this heading structure. In response to the current comment, we have removed the numerous sub-headings and reorganized the Discussion into a continuous narrative flow, retaining only three broad section headings (Structural Determinants, Intermediary Determinants, and Cross-Cutting Factors/Policy Implications) to preserve the manuscript's overall organization while allowing the argument to flow more naturally within each section.

10. Line 578-592: Inserting a sub-header, “Implications for Policy and Practice” may help readers.

Done. This sub-header has been inserted before the relevant paragraph.

11. Lines 594-609: Inserting a sub-header, “Strengths and Limitations” may be helpful for readers.

Done. This sub-header has been inserted (renamed from "Limitations" to "Strengths and Limitations" per the reviewer's suggestion).

12. Lines 610-642: The Conclusion seems rather lengthy. Could it be made more succinct?

Done. The Conclusion has been condensed from four paragraphs to two, retaining the SDH framework reference, key finding, policy recommendations, and the study's place within the mixed-methods design.
