# Supplementary material for: Social determinants of access to reproductive health services among Afghan migrant women in Iran: A qualitative study
Source: PLOS Glob Public Health. 2026 Oct 15;6(10):e0007406. doi: 10.1371/journal.pgph.0007406 (PMC13671505; doi:10.1371/journal.pgph.0007406)
Supplement: Supplementary file 9 [file pgph.0007406.s003.docx]

Review comments

Title: Social Determinants of Access to Reproductive Health Services among Afghan Migrant Women in Iran: A Qualitative Study

Dear Author,

Thank you for submitting this article to PGPH. It raises some pertinent issues worthy of investigation at this time when the world is in peril, and populations continue to be displaced and affected adversely by instability and war, further exacerbating challenges faced by migrants in Iran and elsewhere. Congratulations for a timely piece of research!

I have some suggestions for your consideration and hope you find them useful to strengthen your manuscript and to ensure a high-quality publication as you work towards your PhD.

General comments:

1. Inserting sentence line numbers would greatly help this feedback process.
2. Please double check the entire manuscript for typos and grammatical mistakes.
3. Avoid bullet points and indentation of paragraphs for better flow of the report’s narrative.
4. Please use Headers (formatting) to delineate sections and chapters to ease flow and readability.
5. Rework the table captions and formatting.

Introduction:

1. Please define these key terms as used in the manuscript: Migration; Migrants (and distinguish from refugees); Access; Sexual/Reproductive Health Services; SDH and the WHO SDH framework.
2. In paragraph 1, the number of Afghan refugees is an Arabic symbol, please replace ۳.۴۷ for readability.
3. Great that human right to health and intersectionality are mentioned. However, these appear not to be pursued and further developed in other sections of the manuscript such as analysis (methods) and discussion. Likewise power relations.
4. Although the challenges faced by migrant Afghan women are described, the introduction lacks a compelling statement of the public health problem and its significance. For example, SDH is useful as framework for analyzing/promoting health equity, but no case is made regarding social inequalities or socially-patterned inequalities in (sexual) reproductive health which would answer the question: why focus on Afghan migrant women (yes, they face many challenges, but these challenges aren’t at all unique to them).
5. In paragraph 2, the second sentence appears to suggest that the WHO developed its SDH framework to respond to challenges faced by migrant Afghan women. Please rephrase as the framework seeks to tackle similar challenges but was not necessarily responding (only) to issues faced by migrant Afghan women.
6. Also, please include a citation for the WHO SDH framework in this instance where it is first mentioned. There have been several WHO SDH publications since the CSDH report in 2008 and it would be helpful to know which iteration or specific version is being referenced here to signal the “framework” analysis which follows.
7. Alongside comment #6 above, citations 8&9 included in text are expected to be those related directly to the WHO SDH framework because you are elaborating on the components of the WHO SDH framework. However, publications contained in the reference list are secondary to this and not the primary reference, which should be the actual WHO SDH framework. Please double check that the in-text citation and the reference list back up the text sufficiently by distinguishing whether you are referring to applications of the WHO SDH framework or to the actual (original) framework as published by WHO.
8. It might be helpful to readers if you reproduced the framework in the form of a diagram/figure after a detailed description. Since this framework is core to your work, this might help limit any misunderstanding, here in the introduction or later in the methods/discussion. It might also reassure readers that you are referring to a specific framework.
9. It might be worth considering how relevant in its complete form the framework, or the extent to which the framework can be applied holistically, considering that your study seeks to explore issues around “**access**” while the SDH framework is more expansive and goes beyond access (in fact health service access is one of the many of its facets). Following this reflection, it might be useful to further consider whether you need(ed) to make any iterations or to operationalize the framework for use in your research and if this was already the case (it isn’t clear), to explain how you went about this.
10. Paragraph 4 commences thus, “A qualitative approach is well-suited to explore these issues”. It is not clear why you are justifying the methodology in this section. If it is the case that you are highlighting the knowledge gap, you might want to do so without getting into the merits of qualitative research for your current endeavour. It might also be too early to get into the strategies for trustworthiness. Consider leaving this to either the analysis sections or discussion sections (limitations and strengths).
11. It is useful to see reference to a “comprehensive conceptual framework that integrated multiple pathways..” However, the ensuing analysis in the methods and discussion sections do not sufficiently bring this out, and “intersectionality” lens might come in handy for this.
12. It could be argued that it is not sufficient to fault existing studies for using regression and models suited to high income countries. There is need to highlight the key difference between LMIC/Iran and HICs that make these less suited to studying this public health problem.
13. I notice that you are using SRH and RH interchangeably. It would help to be consistent or to clarify that you indeed intend to use these interchangeably, and why – early in the manuscript.
14. Please rephrase this for better flow: *This represents a key gap: no prior research in Iran has systematically examined the influence of social determinants on Afghan migrant women's access to reproductive health services using a qualitative lens grounded in the SDH model (5,12).* You could omit, “This represents a key gap” which seems redundant. You could also replace “grounded” with a more suitable word to avoid confusion (as some readers might mistake this –and question it – for grounded theory analysis.
15. *The aim—to illuminate these determinants and support authorities in addressing unmet needs and barriers—was achieved via thematic exploration of participants' perspectives, revealing actionable insights for improving equitable SRH access*. Please rewrite for better clarity. You could try: “This study aimed to illuminate the social determinants of….among migrant Afghan women living in Iran”. I would also advise that you replace “thematic” with a better word to avoid mix up with a related analysis method (thematic analysis/thematic content analysis) for readers. It might be helpful to clarify how the research attained its partial aim of “supporting authorities to address unmet needs and barriers” or to leave it out of the study’s aim altogether. For example, having collected data, analysed it and written a report, did you go further to help authorities take action to address identified SDH barriers? If this is reported elsewhere, please mention.
16. While it is useful to explore barriers… it would equally be helpful to have looked at enablers or facilitators, or to identify what’s working that authorities might build upon, or is the situation entirely adverse? Could also help balance conclusions but not mandatory to do. Just a thought.

Methods:

1. “This qualitative study formed part of a doctoral thesis and employed directed content analysis (***insert citation***) guided by the World Health Organization's Social Determinants of Health (SDH) framework (***insert citation***)”. Citations need to be included in the indicated areas for readers to be able to ascertain relevance and congruence, and judge rigour or credibility of analysis by comparing what was done to what ought to have been done. For example, a reader needs to be able to compare the data analysis steps you took in practice against the reference “directed qualitative content analysis”. Did you use the 16-step approach proposed by Assarroudi and colleagues (2018)? There will be other iterations, hence the need to reference the one you adopted for your study.
2. “The analysis focused on structural (e.g., socioeconomic position, migration policies) and intermediary (e.g., material circumstances, psychosocial factors, health behaviors) determinants and their influence on access to reproductive health services among Afghan migrant women in Iran.” Earlier you made the case for having applied the WHO SDH framework through a directed qualitative content analysis. Please clarify whether the framework was used to generate a codebook a priori which was then applied to the data, or not. Clary why the analysis only focused on structural and intermediary factors (as elsewhere you mention other cross-cutting barriers)?
3. Please include a clear rationale for including those who had resided in Tehran for more than 1 year only. Please also justify the inclusion of only 18-45year olds. Why not 15-49, for example?
4. Because of stigma and existing poor access to services for the target study participants, as earlier described, reflect on whether other sampling and recruitment strategies (e.g., snowball or going beyond the health centers) might have yielded a sample with better information power.
5. Theoretical saturation (mostly suited to Grounded Theory approach which relies a lot on the constant comparison process) need not be achieved always, and need not be a goal of this kind of research, I think. You could stop recruitment when you have had enough data for analysis, as well as due to any number of other reasons ranging from the practical (time, finances, insecurity etc). Whatever the case, it might be helpful to provide more detail regarding how you came to the conclusion that no new themes or insights were emerging. What if a tweak in the sampling approach might have invited more themes? As well, the phrase, “no new themes emerged” could confuse readers who may see your analytical approach as thematic analysis. Try and rephrase for better clarity.
6. It might be useful to mention how power relationships were navigated in this study, considering how vulnerable migrant women might be (even though adults), and the recruitment strategy (catching them in the process of seeking services). Also, some appear to have had difficult experiences, if not most. Was any sort of psychosocial relief availed during interviews for those that might have come under distress? Were participants informed that they could pause or stop interviews etc?
7. Include the interview guide as an appendix and clarify whether the same guide was used for migrants and for key informants.
8. Consolidate statements on theoretical saturation. At the moment these are scattered through more than one section.
9. 20 women and 10 key informants eventually included. What was the intended/planned sample size?
10. Data processing and analysis: please state the language of interviews. Also write out this section in prose/narrative paragraph form and eliminate the use of bullet points.
11. Research team and reflexivity: Please see consider a statement on positionality as part of your explication of any biases. (note: bias isn’t always a bad thing!).

Findings:

1. It is not clear why husbands’ socio-demographics are included. It might be useful to mention these more analytically as various categories of findings are presented/discussed.
2. Consider having another go at the analysis with a view to (1) reducing overlap among categories, sub-categories and codes, and (2) paying more fidelity to the SDH framework. You might reduce the numbers of categories, drawing from second order coding (analytical codes) and better focus your findings. It might also be helpful to reconsider which codes fit better under which sub-categories/categories. E.g., item 6- Enhanced Access to Mobile Clinics and Public Health Programs under is listed under code “Social Policy” which falls under the sub-category of “Socio-economic context”, which is fitted under the broader category of “Structural determinants”. Would this fit better elsewhere? Also, is there a good reason why Behavioural factors are separated from Biological factors where considerable overlaps exist as evidenced in participant quotes?
3. Please consider applying Human Rights, Gender, Intersectionality, or Power (and empowerment) lenses which might help enrich the analysis and discussion chapters and yield stronger actionable recommendations. Don’t have to use all lenses although all are congruent with SDH framework, considering the pathways through which SDH lead to deprivations or disparities in access to health.

Conclusion:

1. Consider suggestion #3 above and if useful, use it to strengthen the conclusion. It is quite clear that numerous barriers are constraining access to services. How is this situation tenable? What is the overall impact? Why should the authorities care to do something about it? And What more could be done? Make this section more crisp and concise.

References:

Suggest looking at the following too

1. Solar O, Irwin A. A conceptual framework for action on the social determinants of health. Social Determinants of Health Discussion Paper 2 (Policy and Practice). Available: https://iris.who.int/server/api/core/bitstreams/ca294183-3263-470f-a5fe-8e124ec48c72/content
2. Dahlgren G, Whitehead M. The Dahlgren-Whitehead model of health determinants: 30 years on and still chasing rainbows. Public Health. 2021 Oct;199:20-24. doi: 10.1016/j.puhe.2021.08.009. Epub 2021 Sep 14. PMID: 34534885.
3. Frank J, Abel T, Campostrini S, Cook S, Lin VK, McQueen DV. The Social Determinants of Health: Time to Re-Think? Int J Environ Res Public Health. 2020 Aug 12;17(16):5856. doi: 10.3390/ijerph17165856. PMID: 32806743; PMCID: PMC7459980.
4. Evidence on Social Determinants of Health. World Health Organization 2017. <http://www.who.int/social_determinants/themes/en>.
5. Marmot M. Social Determinants of Health Inequalities. The Lancet 2005; 365(9464): 1099-1104. DOI: https://doi.org/10.1016/S0140-6736(05)71146-6.
6. Krieger N. A Glossary for Social Epidemiology. Journal of Epidemiology and Community Health 2001; 55: 693–700. DOI: <http://dx.doi.org/10.1136/jech.55.10.693>.
7. The Economics of the Social Determinants of Health and Health Inequalities: A Resource Book. World Health Organization 2013. <http://www.who.int/social_determinants/publications/9789241548625/en>
8. Energy: Shared Interests in Sustainable Development and Energy Services. World Health Organization 2013. <http://www.who.int/social_determinants/publications/9789241506199/en>
9. Closing the Health Equity Gap: Policy Options and Opportunities for Action. World Health Organization 2013. <http://apps.who.int/iris/handle/10665/78335>
10. Hidden Cities: Unmasking and Overcoming Health Inequities in Urban Settings. World Health Organization, United Nations Settlements Programme 2010. <http://www.who.int/kobe_centre/publications/hidden_cities2010/en>.
11. Closing the Gap in a Generation: Health Equity Through Action on the Social Determinants of Health: Final Report of the Commission on Social Determinants of Health. World Health Organization 2008. <http://www.who.int/social_determinants/thecommission/finalreport/en>
12. Phelan J et al. Social Conditions as Fundamental Causes of Health Inequalities: Theory, Evidence, and Policy Implications. Journal of Health and Social Behavior 2010; 51(S): S28-40. DOI: <http://doi.org/10.1177/0022146510383498>.
13. Heiman H, Artiga S. Beyond Health Care: The Role of Social Determinants in Promoting Health and Health Equity. Henry J. Kaiser Family Foundation 2015. <http://www.kff.org/disparities-policy/issue-brief/beyond-health-care-the-role-of-socialdeterminants-in-promoting-health-and-health-equity>
