# Supplementary material for: Social determinants of access to reproductive health services among Afghan migrant women in Iran: A qualitative study
Source: PLOS Glob Public Health. 2026 Oct 15;6(10):e0007406. doi: 10.1371/journal.pgph.0007406 (PMC13671505; doi:10.1371/journal.pgph.0007406)
Supplement: Supplementary file 10 [file pgph.0007406.s004.docx]

Dear Reviewers and Editorial Team,

We would like to express our sincere gratitude for the time and effort you dedicated to providing such insightful and constructive feedback on our manuscript. Your thorough review has been instrumental in enhancing the analytical depth and structural clarity of this study. We have carefully considered each of your comments and have revised the manuscript accordingly to address the concerns raised, particularly regarding the theoretical framework and the socio-structural implications of our findings.

Below, we provide a point-by-point response to the comments.

| Comment general | Reviewer’s General Comment Summary | Author’s Response & Action Taken |
| --- | --- | --- |
| 1 | Insert sentence line numbers for easier feedback. | Action Taken: Continuous line numbers have been added throughout the entire manuscript to facilitate the review and tracking of changes. |
| 2 | Double-check for typos and grammatical mistakes. | Action Taken: The entire manuscript has undergone a thorough professional proofreading and grammatical review With AI to ensure clarity and academic rigor. |
| 3 | Avoid bullet points and indentation of paragraphs to improve narrative flow. | Action Taken: All bullet points in the Discussion and Conclusion sections have been converted into cohesive, narrative paragraphs. Paragraph indentations have been adjusted according to the journal’s style guide. |
| 4 | Use Headers (formatting) to delineate sections and chapters. | Action Taken: Professional hierarchical headers have been applied throughout the manuscript to clearly define sections, improving overall flow and readability. |
| 5 | Rework table captions and formatting. | Action Taken: All table captions and formatting have been revised for consistency. Captions are now more descriptive, and tables adhere to the standard APA/journal-specific guidelines. |

Response to Reviewer – Introduction Section

Here is the updated two-column table with responses tailored precisely to the current version of your Introduction:

| Introduction | Reviewer’s Comment | Authors’ Response |
| --- | --- | --- |
| 1 | 1. Please define these key terms as used in the manuscript: Migration; Migrants (and distinguish from refugees); Access; Sexual/Reproductive Health Services; SDH and the WHO SDH framework. | Thank you. We have added explicit definitions in the revised Introduction. “Migration” and the distinction between “migrants” and “refugees” are now clearly defined in paragraph 2. “Access” is defined using Levesque et al.’s five dimensions (paragraph 3). SRH is defined in paragraph 3, and the WHO SDH framework is introduced and explained in paragraph 5 with reference to structural and intermediary determinants. |
| 2 | 2. In paragraph 1, the number of Afghan refugees is an Arabic symbol, please replace ۳.۴۷ for readability. | Corrected. The number now appears as “3.47 million” using standard Western numerals. |
| 3 | 3. Great that human right to health and intersectionality are mentioned. However, these appear not to be pursued and further developed in other sections… power relations. | We have strengthened the integration of human rights, intersectionality, and power relations. These concepts are now explicitly linked and carried through into the Discussion sections. |
| 4 | 4. Although the challenges… the introduction lacks a compelling statement of the public health problem and its significance… why focus on Afghan migrant women. | We have significantly revised paragraphs 4 and 5 to better articulate the public health significance, the magnitude of SRH inequities, and the rationale for focusing on Afghan migrant women in Iran, emphasizing socially patterned inequalities and health equity. |
| 5 | 5. In paragraph 2, the second sentence appears to suggest that the WHO developed its SDH framework to respond to challenges faced by migrant Afghan women. Please rephrase… | Rephrased. The text now clearly states that the WHO SDH framework is applied to understand the challenges faced by Afghan migrant women, without implying it was developed specifically for them. |
| 6 | 6. Also, please include a citation for the WHO SDH framework… which iteration… | Added. The primary citation is now Commission on Social Determinants of Health (2008) – Closing the gap in a generation (reference 11), along with a more recent application reference. |
| 7 | 7. Alongside comment #6… citations 8&9… Please double check… | We have revised the in-text citations and reference list. Citations related to the SDH framework now accurately point to the original WHO documents rather than only secondary sources. |
| 8 | 8. It might be helpful… reproduced the framework in the form of a diagram/figure… | We have included Figure 1 in the revised manuscript showing the WHO SDH framework (with structural and intermediary determinants) as suggested. |
| 9 | 9. It might be worth considering how relevant… operationalize the framework… | We have added a sentence in paragraph 5 explaining how the framework was operationalized for this study, with a specific focus on the five dimensions of access (Levesque) and the interplay between structural and intermediary determinants. |
| 10 | 10. Paragraph 4 commences thus, “A qualitative approach is well-suited…” It is not clear why you are justifying the methodology… | This sentence has been removed from the Introduction. Methodological justification has been moved entirely to the Methods section. |
| 11 | 11. It is useful to see reference to a “comprehensive conceptual framework… intersectionality” lens… | We have further integrated the intersectionality lens in the revised Introduction (paragraph 4) and strengthened its application in the Discussion sections. |
| 12 | 12. It could be argued that it is not sufficient to fault existing studies for using regression… highlight the key difference… | We have expanded this section (paragraph 6) to clearly explain the contextual differences between LMICs (especially Iran under sanctions) and HICs, including legal invisibility of undocumented migrants, economic volatility, and limitations of quantitative models in capturing structural barriers. |
| 13 | 13. I notice that you are using SRH  and RH interchangeably… | We now consistently use “SRH” (Sexual and Reproductive Health) throughout the manuscript. This has been standardized. |
| 14 | 14. Please rephrase this for better flow: “This represents a key gap: no prior research…” | Rephrased for better academic flow as: “To date, no study in Iran has utilized the SDH framework to examine the social determinants influencing Afghan migrant women’s access to reproductive health services.” |
| 15 | 15. The aim—to illuminate these determinants… Please rewrite for better clarity… | The aim has been rewritten for precision and clarity as: “This study aimed to explore the social determinants influencing Afghan migrant women’s access to sexual and reproductive health services in Iran using the WHO SDH framework.” The phrase about “supporting authorities” has been removed from the aim and placed more appropriately in the Implications section. |
| 16 | 16. While it is useful to explore barriers… it would equally be helpful to have looked at enablers or facilitators… | Thank you. Although the main focus was on barriers, we mentioned official free-of-charge PHC policies as a formal facilitator, contrasting it with actual barriers. |

Response to Reviewer – Methods Section

| Comment methods | Reviewer’s Comment | Authors’ Response |
| --- | --- | --- |
| 1 | 1. “This qualitative study formed part of a doctoral thesis and employed directed content analysis… Citations need to be included… Did you use the 16-step approach proposed by Assarroudi and colleagues (2018)?” | Thank you. We have now clearly cited the approach: The study employed directed qualitative content analysis as described by Hsieh and Shannon (2005) and specifically followed the 16-step procedure proposed by Assarroudi et al. (2018). Both references are included, and the text states that this exact procedure was followed. |
| 2 | 2. “The analysis focused on structural… and intermediary…” Please clarify whether the framework was used to generate a codebook a priori… Clarify why the analysis only focused on structural and intermediary factors. | We have clarified this in the “Data Processing and Analysis” subsection. The WHO SDH framework was used deductively to develop an a priori codebook based on structural and intermediary determinants. Both deductive and inductive coding were applied. Cross-cutting issues such as intersectionality, gender, and power relations were integrated within these two main levels as they manifested in the data. |
| 3 | 3. Please include a clear rationale for including those who had resided in Tehran for more than 1 year only. Please also justify the inclusion of only 18-45 year olds. Why not 15-49, for example? | We have added the rationale in the “Study Design and Sampling” subsection. Residence in Tehran for at least one year ensures sufficient exposure to the urban environment and reduces bias from very recent arrivals. The age range 18–49 years was chosen to cover the full reproductive age span of adult women while excluding adolescents (under 18, due to ethical and legal requirements for parental consent) and focusing on women before the typical onset of menopause and age-related comorbidities. This range is more suitable for our focused SRH study than the broader 15–49 demographic range used in large surveys. |
| 4 | 4. Because of stigma… reflect on whether other sampling and recruitment strategies (e.g., snowball or going beyond the health centers) might have yielded a sample with better information power. | We have addressed this by expanding recruitment beyond health centers to municipal recreational and educational hubs (“Shahr-banoo” centers) and local NGOs. This strategy successfully reached undocumented women and non-users of formal services, thereby increasing the diversity and information power of the sample. |
| 5 | 5. Theoretical saturation… rephrase for better clarity. | We have revised the text and now use the term “data saturation” (defined as the point at which additional interviews yielded no new codes or conceptual insights relevant to the research framework). All mentions have been consolidated into one clear paragraph in the sampling section. |
| 6 | 6. It might be useful to mention how power relationships were navigated… psychosocial relief… right to pause or stop. | We have expanded the “Research Team and Reflexivity” and Ethical Considerations sections. The lead researcher (a midwife) was mindful of power imbalances. Participants were informed they could pause or stop the interview at any time. An Afghan midwifery student acted as a peer facilitator to reduce power differentials and build trust. Referral information for psychosocial support was available if needed. |
| 7 | 7. Include the interview guide as an appendix and clarify whether the same guide was used for migrants and for key informants. | The semi-structured interview guide for Afghan migrant women is provided as Appendix 1. A tailored version for key informants was also used. This has been clarified in the Data Collection subsection. |
| 8 | 8. Consolidate statements on theoretical saturation. At the moment these are scattered through more than one section. | All statements related to data saturation have been consolidated into a single clear paragraph in the “Study Design and Sampling” section. |
| 9 | 9. 20 women and 10 key informants eventually included. What was the intended/planned sample size? | We sincerely appreciate the reviewer’s specific inquiry regarding our sample size.  Initially, we planned to recruit 25 to 30 Afghan migrant women and 12 to 15 key informants (a total of approximately 37 to 45 participants). This target was established based on the principles of qualitative sample size estimation, considering the heterogeneity of the Afghan migrant population (in terms of age, duration of residency, socioeconomic status, and residential area) and the need for sufficient depth and breadth of information required for a Directed Content Analysis guided by the SDH framework.  Ultimately, 20 Afghan migrant women and 10 key informants (totaling 30 participants) were included in the study. Data collection was concluded upon reaching informational redundancy; that is, when new interviews no longer yielded any new codes or insights regarding the structural and intermediary determinants of access to reproductive health services. While the final sample size was slightly lower than initially projected, it was deemed sufficient to achieve the research objectives and ensure a comprehensive analysis of the data." |
| 10 | 10. Data processing and analysis: please state the language of interviews. Also write out this section in prose/narrative paragraph form… | The section has been fully rewritten in narrative paragraph form. All interviews with Afghan women were conducted in Persian or Dari. An Afghan midwifery student served as a peer facilitator to ensure linguistic and cultural accuracy. |
| 11 | 11. Research team and reflexivity: Please consider a statement on positionality as part of your explication of any biases. | We have substantially expanded the “Research Team and Reflexivity” subsection with a detailed positionality statement. The lead researcher’s background as an Iranian midwife was both an advantage (building trust on SRH topics) and required active reflexivity to manage power imbalances. |

| Comment results | Reviewer’s Comment Summary | Author’s Response & Action Taken | Related Section |
| --- | --- | --- | --- |
| 1 | The rationale for including husbands’ socio-demographics is unclear. They should be presented more analytically. | Husbands’ education and occupation have been analytically integrated into the Social Class section. These variables are now explicitly linked to gender norms, household decision-making power, and their direct impact on women’s autonomy in seeking reproductive care. | Results (Social Class) + Discussion |
| 2 | Reduce overlap among categories/codes and increase fidelity to the WHO-SDH framework. Reconsider placements (e.g., Mobile Clinics) and merge Behavioral/Biological factors. | 1. Behavioral and Biological factors have been merged into a single category: "Bio-behavioral Factors" to reflect their inherent overlap.    2. "Mobile Clinics" was moved from Social Policy to Health System Factors (Intermediary Determinants).    3. Social and Public Policy sections were refined to eliminate redundancy and ensure a clearer hierarchical structure aligned with the SDH framework. | Results (Table 3 & all sub-sections) |
| 3 | Apply Human Rights, Gender, Intersectionality, or Power lenses to enrich the analysis and recommendations. | hank you for this valuable comment.  The entire qualitative analysis was conducted based on the World Health Organization’s Social Determinants of Health (SDH) framework. We used a directed qualitative content analysis approach, with an a priori codebook derived directly from the structural and intermediary determinants of the WHO SDH framework. All identified determinants were mapped under the relevant categories of this framework.  In the revised manuscript, we have re-organized the Results section and Table 2 (Summary of Findings) so that the most prominent and frequently mentioned issues are clearly placed under their specific sub-categories within the structural and intermediary determinants. The final categorization and interpretation of themes were finalized after thorough team discussion and consensus to ensure accuracy and coherence with the theoretical framework.. | Results & Discussion (All sections) |

| Comment Conclusion | Reviewer’s Comment Summary | Author’s Response & Action Taken | Related Section |
| --- | --- | --- | --- |
| 1 | Strengthen the conclusion using analytical lenses (Power, Gender, Intersectionality). Address why the situation is untenable, its overall impact, why authorities should act, and provide concise recommendations. | 1. The current landscape was explicitly defined as "untenable" due to the gap between policy provisions (SIB system) and actual utilization.    2. Using an Intersectional lens, we analyzed how "legal invisibility" and patriarchal norms create a "vulnerability trap" for women.    3. We addressed the authorities' concern by framing migrant health as a matter of "public health security" and "epidemiological safety," highlighting that exclusion increases long-term emergency costs for the host country.    4. Recommendations were made more crisp and concise, focusing on decoupling health from legal status, male-inclusive models, and mobile infrastructure. | Conclusion |

Finally, we would like to express our deepest appreciation for the constructive dialogue during this review process. Given the challenging and unique circumstances of conducting academic research in Iran at this time, it is profoundly meaningful to us that our work has been recognized and scrutinized with such care. Your feedback has not only improved this manuscript but has also strengthened our commitment to advancing health equity for marginalized populations under difficult conditions. We hope the revised version meets your expectations for publication.

Sincerely,

Zahra Bayat Jozani and Co-authors
