# Supplementary material for: Social determinants of access to reproductive health services among Afghan migrant women in Iran: A qualitative study
Source: PLOS Glob Public Health. 2026 Oct 15;6(10):e0007406. doi: 10.1371/journal.pgph.0007406 (PMC13671505; doi:10.1371/journal.pgph.0007406)
Supplement: Supplementary file 11 [file pgph.0007406.s005.docx]

**Dear Reviewers and Editorial Team,**

**We would like to express our sincere gratitude for the time and effort you dedicated to providing such insightful and constructive feedback on our manuscript. Your thorough review has been instrumental in enhancing the analytical depth and structural clarity of this study.**

**We are truly sorry that the valuable comments from Reviewer #1 were inadvertently overlooked in the previous revision round. We have now carefully addressed all comments from both reviewers, particularly those raised by Reviewer #1. We apologize for this oversight and appreciate your understanding and the opportunity to resubmit a fully revised version.**

**We have carefully considered each of your comments and have revised the manuscript accordingly to address the concerns raised, particularly regarding the theoretical framework, socio-structural implications, ethnic diversity, and future research directions.**

**Below, we provide a point-by-point response to the comments.**

**eviewer #1:** While the authors have responded to the second reviewer point by point, they have completely ignored my comments. I had made the following comments and i suggest in both Discussions and conclusions, the authors specifically address these areas:

1. An area not covered in the study is the ethnic background of women from Afghanistan itself. Depending on where they come from for example, Hazra community or from Tajik or Pashtun or Uzhbek, the response and behavior to seek RH varies. This has not been mentioned by the authors.

We sincerely thank Reviewer #1 for this valuable and insightful comment. In response, ethnic background has been added as a demographic variable to Table 1, reflecting the composition of the sample: the majority of participants identified as Hazara (n=17, 85%), with a smaller proportion identifying as Pashtun (n=3, 15%). All participants were Muslim, with Hazara participants identifying as Shia and Pashtun participants as Sunni.

However, we acknowledge that the interview guide did not include specific questions addressing how ethnic or religious identity influenced reproductive health-seeking behavior. Furthermore, since all participants shared the same religious faith (Islam), religion did not emerge as a differentiating factor in the analysis. With regard to ethnicity, the linguistic dimension — namely, differences between Dari and Pashto speakers — was observed as a practical barrier during data collection and is reflected in the findings; however, a systematic comparative analysis across ethnic groups was not feasible within the scope of this study.

We fully agree with the reviewer that intra-Afghan ethnic diversity is a meaningful dimension that may shape health-seeking behavior in distinct ways. Accordingly, this has been explicitly acknowledged in the Limitations section of the revised manuscript, with a clear recommendation that future studies employ purposive sampling strategies ensuring adequate representation across Afghan ethnic communities to enable rigorous comparative analysis.

2. I would also suggest that they authors note clearly how they themselves would use the analysis and what kind of future studies will be planned on their sites. Additional analysis in terms of variation by age, ethinicity, reasons for migration (such as marriage into a Iranian family vs. refugee or undocumented status), prior knowledge and care seeking behaviour when they were in Afghanistan are areas that I hope will be covered in a larger, broader study in future.

The authors need to clearly note how they plan to use this small study further.

We thank the reviewer for this constructive and forward-looking suggestion. We fully agree that explicitly articulating the research team's plans for utilizing these findings and outlining future research directions is essential for contextualizing the contribution of this study.

This qualitative study constitutes the first phase of a larger sequential exploratory mixed-methods doctoral research program conducted at Tehran University of Medical Sciences. The themes, determinants, and conceptual pathways identified in this qualitative phase have directly informed the development of a quantitative instrument, which was subsequently administered to a larger sample of Afghan migrant women in Tehran. The quantitative phase employed path analysis and structural equation modeling to test the causal relationships between the social determinants identified here and actual reproductive health service utilization. The integrated findings of both phases will further inform the development of a context-specific policy package aimed at improving equitable access to reproductive health services for Afghan migrant women in Iran.

We fully concur with the reviewer that future independent studies should expand this line of inquiry to examine variation by age group, ethnic background, and reason for migration — distinguishing, for example, women who migrated through marriage into an Iranian family from those displaced as refugees or residing as undocumented economic migrants. Additionally, investigating women's prior reproductive health knowledge and care-seeking behavior before migration to Iran would provide critical insight into how pre-migration experiences shape post-migration health utilization patterns. These dimensions were beyond the scope of the current qualitative phase but represent important priorities for the broader research agenda in this field. We have incorporated these points explicitly into the Limitations and Conclusion sections of the revised manuscript.

| Comment general | Reviewer’s General Comment Summary | Author’s Response & Action Taken |
| --- | --- | --- |
| 1 | Insert sentence line numbers for easier feedback. | Action Taken: Continuous line numbers have been added throughout the entire manuscript to facilitate the review and tracking of changes. |
| 2 | Double-check for typos and grammatical mistakes. | Action Taken: The entire manuscript has undergone a thorough professional proofreading and grammatical review With AI to ensure clarity and academic rigor. |
| 3 | Avoid bullet points and indentation of paragraphs to improve narrative flow. | Action Taken: All bullet points in the Discussion and Conclusion sections have been converted into cohesive, narrative paragraphs. Paragraph indentations have been adjusted according to the journal’s style guide. |
| 4 | Use Headers (formatting) to delineate sections and chapters. | Action Taken: Professional hierarchical headers have been applied throughout the manuscript to clearly define sections, improving overall flow and readability. |
| 5 | Rework table captions and formatting. | Action Taken: All table captions and formatting have been revised for consistency. Captions are now more descriptive, and tables adhere to the standard APA/journal-specific guidelines. |
| Introduction | Reviewer’s Comment | Author’s Response & Location of Change |
| 1 | Define key terms: Migration, Migrants vs. Refugees, Access, SRH, and SDH. | Paragraphs 1, 2, 3, & 5: Formal definitions have been integrated throughout the introduction. |
| 2 | Replace the Arabic symbol "۳.۴۷" with English numerals. | Paragraph 1: The figure has been corrected to "3.47 million." |
| 3 | Develop concepts of human rights, intersectionality, and power relations. | Paragraphs 3, 4, & 6: These concepts are now explicitly linked to structural barriers and inequity. |
| 4 | Provide a compelling statement on socially-patterned inequalities. | Paragraph 4: A new section was added to explain the socially-patterned health inequities in the Iranian context also we add in discussion |
| 5 | Clarify that the WHO-SDH framework is a general tool, not specific to Afghan women. | Paragraph 5: Phrasing was revised to present the framework as a universal analytical tool |
| 6 | Include the original citation for the WHO-SDH framework (Solar & Irwin, 2010). | Paragraph 5: The original citation (Solar & Irwin, 2010) has been added at the first mention. |
| 7 | Distinguish between the original framework and secondary applications. | Paragraphs 5 & 6: Citations have been separated to distinguish the original framework from secondary studies. |
| 8 | Reproduce the SDH framework as a diagram/figure. | Paragraph 5: Added a reference to Figure 1, which illustrates the conceptual framework of the study. |
| 9 | Explain how the SDH framework was operationalized for "Access." | Paragraph 5: We clarified how SDH determinants map onto Levesque’s dimensions of access. |
| 10 | Remove methodology justification (qualitative approach) from the Introduction. | Deleted: The sentences justifying the qualitative method were removed and moved to the Methods section. |
| 11 | Enhance the "intersectionality lens" in the analysis. | Paragraph 4: Added a detailed sentence on how legal status, education, and gender intersect. The intersectionality lens, with a specific focus on the intersection of “being a woman, being a migrant, and patriarchy,” has been fully expanded into the Discussion section. |
| 12 | Highlight differences between LMICs (Iran) and HICs regarding model suitability. | Paragraph 6: Discussed structural idiosyncrasies of Iran, including economic sanctions and legal invisibility. |
| 13 | Be consistent with SRH vs. RH terminology. | Entire Text: The manuscript now consistently uses SRH throughout. |
| 14 | Rephrase the gap statement and remove "grounded" to avoid confusion. | Paragraph 7: The phrase "key gap" was removed, and "grounded" was replaced with "grounded in" or "guided by". |
| 15 | Clarify the study aim and avoid the word "thematic." | Paragraph 7: The word "thematic" was removed, and the aim was rephrased for better clarity. |
| 16 | Identify enablers/facilitators to balance the conclusions. | Paragraph 4: Mentioned official free-of-charge PHC policies as a formal facilitator, contrasting it with actual barriers. |
| Methods | Reviewer’s Comment | Author’s Response & Action Taken |
| M1 | Provide specific citations for the analysis method and framework. | Action Taken: Citations for Hsieh & Shannon (2005) for directed content analysis and Assarroudi et al. (2018) for the 16-step analytical process have been included. The foundational citation for the WHO-SDH framework (Solar & Irwin, 2010) was also added. |
| M2 | Clarify the use of an *a priori* codebook and focus of analysis. | Action Taken: We clarified that an *a priori* codebook was developed based on the WHO-SDH framework. The analysis followed a hybrid approach, integrating deductive coding (framework-based) and inductive coding to capture emergent themes such as power relations. |
| M3 | Rationale for inclusion criteria (Age 18–49 and 1-year residency). | Action Taken: The age range (18–49) targets women of reproductive age while ensuring legal adulthood for informed consent. The one-year residency ensures that participants have had sufficient interaction with the Iranian health system to provide meaningful insights. |
| M4 | Reflection on diverse sampling beyond health centers. | Action Taken: Recruitment was expanded to include municipal educational and recreational hubs (Shahr-banoo) and NGOs. This allowed us to reach "non-users" and women living in legal invisibility who do not typically frequent formal health centers. |
| M5 | Clarify "Saturation" and rephrase to avoid confusion. | Action Taken: The term "Theoretical Saturation" has been replaced with "Data Saturation". We defined this as the point where additional interviews yielded no new codes or conceptual insights relevant to the research framework. |
| M6 | Navigation of power relationships and participant distress. | Action Taken: To mitigate power imbalances, an Afghan peer facilitator assisted in recruitment. Locations were chosen based on participant comfort. The lead researcher's midwifery background facilitated a supportive environment, and ethical protocols were in place to address any potential distress. |
| M7 | Include the interview guide as an appendix. | Action Taken: The semi-structured interview guide has been provided as Appendix 2, with specific questions tailored for both migrant women and key informants. |
| M8 | Consolidate statements on theoretical saturation. | Action Taken: All details regarding data saturation and sample size determination have been consolidated into the "Study Design and Sampling" section for better logical flow. |
| M9 | State the language of interviews and formatting. | Action Taken: The methodology now explicitly states that interviews were conducted in Persian and Dari. All bulleted lists have been converted into a cohesive narrative (prose) format as requested. |
| M10 | Include a statement on Positionality and bias. | Action Taken: A dedicated Positionality statement was added. We discussed how the lead researcher's background as a midwife served as a 'positional advantage,' fostering trust and clinical rapport while using reflexive journaling to manage potential biases. |

| Comment results | Reviewer’s Comment Summary | Author’s Response & Action Taken | Related Section |
| --- | --- | --- | --- |
| 1 | The rationale for including husbands’ socio-demographics is unclear. They should be presented more analytically. | Husbands’ education and occupation have been analytically integrated into the Social Class section. These variables are now explicitly linked to gender norms, household decision-making power, and their direct impact on women’s autonomy in seeking reproductive care. | Results (Social Class) + Discussion |
| 2 | Reduce overlap among categories/codes and increase fidelity to the WHO-SDH framework. Reconsider placements (e.g., Mobile Clinics) and merge Behavioral/Biological factors. | 1. Behavioral and Biological factors have been merged into a single category: "Bio-behavioral Factors" to reflect their inherent overlap.    2. "Mobile Clinics" was moved from Social Policy to Health System Factors (Intermediary Determinants).    3. Social and Public Policy sections were refined to eliminate redundancy and ensure a clearer hierarchical structure aligned with the SDH framework. | Results (Table 3 & all sub-sections) |
| 3 | Apply Human Rights, Gender, Intersectionality, or Power lenses to enrich the analysis and recommendations. | An Intersectional lens and Power Relations framework were applied throughout the analysis. We specifically examined how legal status (power) intersects with gender norms and social class to create "compounded disadvantages" for the most vulnerable subgroups, such as illiterate women in undocumented families. | Results & Discussion (All sections) |

| Comment Conclusion | Reviewer’s Comment Summary | Author’s Response & Action Taken | Related Section |
| --- | --- | --- | --- |
| 3 | Strengthen the conclusion using analytical lenses (Power, Gender, Intersectionality). Address why the situation is untenable, its overall impact, why authorities should act, and provide concise recommendations. | 1. The current landscape was explicitly defined as "untenable" due to the gap between policy provisions (SIB system) and actual utilization.    2. Using an Intersectional lens, we analyzed how "legal invisibility" and patriarchal norms create a "vulnerability trap" for women.    3. We addressed the authorities' concern by framing migrant health as a matter of "public health security" and "epidemiological safety," highlighting that exclusion increases long-term emergency costs for the host country.    4. Recommendations were made more crisp and concise, focusing on decoupling health from legal status, male-inclusive models, and mobile infrastructure. | Conclusion |

Finally, we would like to express our deepest appreciation for the constructive dialogue during this review process. Given the challenging and unique circumstances of conducting academic research in Iran at this time, it is profoundly meaningful to us that our work has been recognized and scrutinized with such care. Your feedback has not only improved this manuscript but has also strengthened our commitment to advancing health equity for marginalized populations under difficult conditions. We hope the revised version meets your expectations for publication.

**Sincerely,**

**Zahra Bayat Jozani and Co-authors**
