# Supplementary material for: Social determinants of access to reproductive health services among Afghan migrant women in Iran: A qualitative study
Source: PLOS Glob Public Health. 2026 Oct 15;6(10):e0007406. doi: 10.1371/journal.pgph.0007406 (PMC13671505; doi:10.1371/journal.pgph.0007406)
Supplement: Supplementary file 12 [file pgph.0007406.s006.docx]

PGPH-D-26-00223R2
Social Determinants of Access to Reproductive Health Services among Afghan Migrant Women in Iran: A Qualitative Study
PLOS Global Public Health

Subject: Re: PGPH-D-26-00223R2 - Revised Manuscript and Response to Reviewers

Dear Editor,

Thank you very much for the opportunity to revise our manuscript, "Social Determinants of Access to Reproductive Health Services among Afghan Migrant Women in Iran: A Qualitative Study" (PGPH-D-26-00223R2). We are grateful to you and to the reviewers for the careful, constructive feedback, which has helped us substantially strengthen the manuscript.

In the following, we provide our detailed, point-by-point response to each of the reviewers' comments.

As requested, we have also attached two versions of the revised manuscript:

1. A marked-up version highlighting all changes made (Revised Manuscript with Track Changes)

2. A clean version without tracked changes (Manuscript)

We hope these revisions satisfactorily address the reviewers' concerns and that the manuscript is now suitable for publication in PLOS Global Public Health. We remain available for any further clarification you or the reviewers may need.

Thank you again for your time and consideration.

Sincerely,

Zahra Bayat Jozani

On behalf of all co-authors

**A) Reviewer #2 Comments**

| **#** | **Reviewer** | **Reviewer Comment** | **Status** | **Change Made / Remaining Note** |
| --- | --- | --- | --- | --- |
| 1 | Reviewer 2 | Shorten the abstract (over 382 words; should be under 300) | Addressed | Abstract is now approximately 300 words (at the allowed limit). |
| 2 | Reviewer 2 | Replace "autonomy" with "agency" (lines 39-40) | Addressed | The word "agency" is used in the abstract. |
| 3 | Reviewer 2 | Change "migrant specific" to "migrant sensitive" (lines 43-44) | Addressed | The phrase "migrant-sensitive" is used in the abstract and conclusion. |
| 4 | Reviewer 2 | Clarify "triad of vulnerability" in the main text (line 46) | Addressed | A new interpretive paragraph was added in the Discussion section, referencing the Table 3 findings. |
| 5 | Reviewer 2 | Remove the repetition of "enhance service equity, reduce health disparity" (line 51) | Addressed | The repeated phrase was removed and changed to "enhance service equity and lower long-term healthcare costs." |
| 6 | Reviewer 2 | Add "health equity" or "health disparities" to the keywords (lines 53-54) | Addressed | The phrase "Health Equity" was added. |
| 7 | Reviewer 2 | Replace the year 2013 with numbered citation style (line 73) | Addressed | The in-text citation no longer appears as a year; it remains only in the reference list. |
| 8 | Reviewer 2 | Remove "is based in" and replace with "use" (line 87) | Addressed |  |
| 9 | Reviewer 2 | Change "This study" from capital T to lowercase t (line 90) | Addressed |  |
| 10 | Reviewer 2 | Remove "conversely" and replace with a more appropriate connective (line 93) | Addressed | The word "conversely" was not found in the text; it appears to have been replaced. |
| 11 | Reviewer 2 | Remove "idiosyncrasies" and replace with "determinants" (lines 103-104) | Addressed |  |
| 12 | Reviewer 2 | Add "using an intersectionality lens" to the study aim (line 110) | Addressed |  |
| 13 | Reviewer 2 | Insert the word "health" between "reproductive" and "services" (line 111) | Addressed |  |
| 14 | Reviewer 2 | Standardize font size throughout the paragraphs | Addressed |  |
| 15 | Reviewer 2 | Provide a more detailed explanation of rigour/trustworthiness measures rather than a simple list (lines 154-160) | Addressed | A detailed paragraph on Credibility, Dependability, Confirmability, and Transferability was added; however, the "Trustworthiness" heading is mistakenly repeated twice in a row and one should be removed. |
| 16 | Reviewer 2 | Provide a more balanced reflection on the researcher's positionality and neutrality (Research Team and Reflexivity), given the absence of clinical interaction during the study (lines 161-167) | Addressed | New text was added explaining the absence of clinical interaction and bias-mitigation measures; however, the earlier paragraph on the researcher's midwifery background is repeated almost verbatim a second time and the duplication needs to be removed. |
| 17 | Reviewer 2 | Table 3 - Macroeconomic Policy: the code "high cost of services" seems misplaced | Addressed | This code was moved to the Accessibility & Health Service Delivery sub-category (Financial Access). |
| 18 | Reviewer 2 | Table 3 - Social Class: rewrite the code "job" as "employment/occupation" | Addressed |  |
| 19 | Reviewer 2 | Table 3 - Public Policy: revise overlapping codes and add more specific public-policy examples | Addressed | New, more specific codes were added (subsidized insurance, low-cost housing, legal restrictions on SRH services, registration without functional follow-through). |
| 20 | Reviewer 2 | Table 3 - Material Circumstances: separate the "housing quality" and "nutrition" codes | Addressed | Two distinct codes (Housing quality/density and Nutrition and food security) have been retained. |
| 21 | Reviewer 2 | Table 3 - capitalize the "H" in "Accessibility and Health Service Delivery" | Addressed |  |
| 22 | Reviewer 2 | Table 3 - add codes for financial, geographical, and informational access | Addressed | Financial Access and Geographical Access were clearly added; Service Availability and Facility Sensitivity were also added. |
| 23 | Reviewer 2 | Table 3 - add dimensions of service quality (Timeliness, Safety, Effectiveness, Patient-centeredness, Integration, Efficiency) | Addressed | Only two dimensions (Patient-Centeredness/Responsiveness and Cultural Safety/Effectiveness) were added; the dimensions Timeliness, Safety, Service Integration, and Efficiency were not extracted from the interview data. |

**B) Reviewer #3 Comments**

| **#** | **Reviewer** | **Reviewer Comment** | **Status** | **Change Made / Remaining Note** |
| --- | --- | --- | --- | --- |
| 1 | Reviewer 3 | Clarify whether inductive themes were generated outside the SDH framework, whether any data were inconsistent with the framework, and how new concepts were handled | addressed | Coding began deductively with an *a priori* codebook derived from the WHO-SDH framework's top-level structural and intermediary categories. Two *a priori* sub-categories under the original Socioeconomic Position domain — Gender and Ethnicity/racism — were not distinguishable as independent themes in participants' accounts and were therefore not retained as separate headers, rather than being artificially forced onto unsupportive data. Within the retained categories, all sub-categories and final codes were generated inductively, directly from the transcripts. Several inductively derived codes captured content with no direct equivalent in the original WHO-SDH indicators — for example, preference for home birth, fear of arrest and living secretly, delay in care-seeking due to deportation fears, and the "register-and-wait" pattern of unfulfilled policy commitments (Table 3). Whenever an emerging code did not clearly correspond to an existing top-level category, it was discussed in team meetings to determine whether it constituted a genuinely new domain or a nuance of an existing one; in this dataset, all such codes were judged by consensus to fit within the existing three-level structure (structural, intermediary, and cross-cutting determinants), and no additional top-level category was required. |
| 2 | Reviewer 3 | Clarify how data saturation was assessed: how it was measured, who determined it, whether code saturation or meaning saturation was achieved, and after which interview it occurred | Addressed | A definition of data saturation was added. |
| 3 | Reviewer 3 | The Discussion section is disproportionately long and mostly reviews prior literature rather than interpreting the findings; the study's unique contribution, how it extends prior knowledge, and its policy implications should be more strongly highlighted | Partially addressed | Several new interpretive/synthesizing paragraphs were added (triad of vulnerability, the policy-instrument gap, the cost implications of services), and a policy-implications paragraph was added. |

Reviewer #3: This manuscript is timely, policy-relevant, and well aligned with the aims of PLOS Global Public Health. The qualitative design is generally appropriate, and the manuscript demonstrates several methodological strengths. However, several issues remain regarding methodological transparency, analytical depth, and interpretation of findings before the manuscript can be considered for publication.
1-The manuscript uses directed qualitative content analysis based on the WHO SDH framework. However, the analysis appears largely deductive.The authors should explicitly clarify:
• Were any inductive themes generated outside the SDH framework?
• Were any data inconsistent with the framework?
• How were new concepts handled during coding?
Otherwise the study risks appearing as merely classifying data into predetermined categories instead of generating qualitative insight.

**Response to Reviewer #3, Comment 1:**

We thank the reviewer for raising this important point, and we clarify our approach below.

The WHO-SDH framework was applied deductively only at the top level — its three broad domains (structural, intermediary, and cross-cutting determinants) served as the *a priori* scaffold for analysis, consistent with our aim of testing and contextualizing this framework for the Iranian setting and identifying the specific factors shaping reproductive health service access, which will subsequently inform a quantitative validation phase and a localized policy package. Within that scaffold, however, all sub-categories and final codes were generated inductively, directly from participants' own accounts, rather than being fixed in advance.

This inductive process yielded several final codes with no direct equivalent in the generic WHO-SDH indicator set — for example, a strong preference for home birth, the facilitating role of mobile clinics in reaching hard-to-access populations, and the effect of housing/property-ownership status on reproductive health service use (Table 3). These context-specific codes demonstrate that our use of the framework was not a mechanical exercise in classification, but an empirically grounded process of adapting a generic model to the lived realities of Afghan migrant women in Iran.

Regarding data inconsistent with the framework: two *a priori* sub-categories under the original Socioeconomic Position domain — gender and ethnicity/racism — could not be empirically distinguished as independent themes in our data and were therefore not retained as separate headers. Rather than force these onto unsupportive data, we allowed the coding structure to reflect what participants actually reported.

Finally, whenever an emerging code did not clearly correspond to an existing top-level category, it was discussed collectively by the research team to determine whether it represented a nuance of an existing category or warranted a genuinely new domain. In this dataset, all such codes were judged, by consensus, to fit within the existing three-level structure, and no additional top-level category was required. We believe this iterative, team-based process — moving between the deductive framework and inductively generated codes — is precisely what distinguishes directed content analysis from simple classification, and we have found it to generate genuine analytic insight rather than merely confirming a predetermined structure.

2-The manuscript states that interviews continued until saturation.
However, it does not explain:
• how saturation was assessed;
• who determined saturation;
• whether code saturation or meaning saturation was achieved;
• after which interview saturation occurred.
A more transparent description consistent with current qualitative reporting standards is recommended

**Response to Reviewer #3, Comment 2:**

We thank the reviewer for this important observation. The relevant text in the "Study Design and Sampling" section has been revised and clarified accordingly.

Sampling continued until data saturation was reached in both participant groups. Among the 20 Afghan migrant women, code saturation — the point at which no new codes emerged from the data — was reached after the 17th interview; three subsequent interviews were conducted solely to confirm meaning saturation, i.e., to ensure that no further conceptual depth or variation emerged within the existing codes. Among the 10 key informants, code saturation was reached after the 8th interview, and two additional interviews were conducted for the same confirmatory purpose.

Coding was performed independently by two coders — the corresponding author as first coder and the first author as second coder — and the final determination of saturation was confirmed through discussion and consensus with the entire research team, based on comparison of successive versions of the codebook.

3-The Discussion occupies a disproportionately large part of the manuscript.
Several paragraphs mainly summarize previous literature rather than interpreting the present findings.
Greater emphasis should be placed on:
• what this study uniquely contributes;
• how findings extend previous knowledge;
• implications for policy.

**Response to Reviewer #3, Comment 3:**

We thank the reviewer for this observation. Two new paragraphs were added to the Discussion section to more clearly foreground the study's unique contribution, how it extends prior knowledge, and its policy implications.

**1. "Unique contribution" paragraph** — inserted immediately before the "Structural Determinants of Health" heading, making two specific claims. First, to our knowledge, this is the first application of the WHO-SDH framework through an explicit intersectionality lens to reproductive health access among Afghan migrant women, showing empirically how legal, gender, and cultural determinants interact multiplicatively rather than merely co-occurring. Second, the analysis surfaces two context-specific mechanisms not captured by the generic WHO-SDH indicators: the "register-and-wait" phenomenon (formal service enrollment that does not translate into functional access) and "information poverty," which links public policy, digital access, and health-seeking behavior.

**2. "Policy implications" paragraph** — inserted immediately before the "Limitations" section, offering four concrete, findings-based recommendations: (a) extending basic reproductive health entitlements independent of documentation status; (b) pairing enrollment mechanisms with functional referral pathways and subsidized insurance (to address the "register-and-wait" gap); (c) investing in multilingual digital health platforms and peer-based education (to address information poverty); and (d) integrating intimate partner violence screening into existing migrant-serving primary care contacts.

**3. Trimming of literature-recitation sentences** — two purely summarizing sentences (in the Macroeconomic Policy and Living Conditions subsections) that added no new interpretation were removed or condensed, shifting the overall tone of the Discussion from literature review toward interpretation of findings — with no citations removed.
