# Supplementary material for: Social determinants of access to reproductive health services among Afghan migrant women in Iran: A qualitative study
Source: PLOS Glob Public Health. 2026 Oct 15;6(10):e0007406. doi: 10.1371/journal.pgph.0007406 (PMC13671505; doi:10.1371/journal.pgph.0007406)
Supplement: Supplementary file 13 [file pgph.0007406.s007.docx]

**PLOS Global Public Health**

Social Determinants of Access to Reproductive Health Services among Afghan Migrant Women in Iran: A Qualitative Study **R3**

Reviewer comments

Dear Zahra and co-authors,

Thanks for sharing a revised version of your manuscript. Just a few minor comments to conclude.

1. Line 45: delete extra full stop at the end of the sentence
2. Line 70: Insert space after the full stop “(6).According...”
3. Line 72: Insert a numbered citation for the reference: “…Levesque et al…”
4. Lines 75-77: Rewrite sentence to clarify what is meant by “officially”. Is it by policy decree or some national law, or by verbal commitment of national officials?
5. Lines 116-117: You may use acronyms for WHO and SDH, earlier introduced in the manuscript rather than writing again in full and inserting acronym.
6. Line 159: Separate the sub-heading and the sentence: Start the narrative on Trustworthiness on a new paragraph
7. Line 190: “Findings:..” Delete the colon after “Findings”
8. Line 371: “Discussion:” Delete colon after Discussion.
9. Lines 390-609: Would be better to not have too many sub-headings and to let the discussion flow. Throughout the discussion, draw out contrasts, besides the similarities from other studies, in their findings and methods, as well as settings. It is not always apparent in what settings the studies being cited originate (whether Afghanistan or elsewhere). Including the country contexts would help readers make informed comparisons. Also, critique the studies’ methods to enable readers judge the strength of evidence and the conclusions being compared with the present study.
10. Line 578-592: Inserting a sub-header, “Implications for Policy and Practice” may help readers.
11. Lines 594-609: Inserting a sub-header, “Strengths and Limitations” may be helpful for readers.
12. Lines 610-642: The Conclusion seems rather lengthy. Could it be made more succinct?
