# Supplementary material for: Social determinants of access to reproductive health services among Afghan migrant women in Iran: A qualitative study
Source: PLOS Glob Public Health. 2026 Oct 15;6(10):e0007406. doi: 10.1371/journal.pgph.0007406 (PMC13671505; doi:10.1371/journal.pgph.0007406)
Supplement: Supplementary file 14 [file pgph.0007406.s009.docx]

Review comments on PGPH-D-26-00223R4

Dear Zahra and colleagues,

Thanks for sharing a revised manuscript addressing previous review comments. You’re nearly there.

73-79: This sentence is very long.

For Afghan migrant women in Iran, SRH status is shaped by a complex intersection of sociodemographic, cultural, and structural factors Although Iranian law — including constitutional provisions on the right to health and social security (Articles 29 and 43) and the mandatory insurance clause for foreign nationals under the Sixth Five-Year Development Plan (Article 70) — has been interpreted as establishing a legal commitment to provide primary healthcare, including maternal and child health services, to Afghans regardless of legal status, a significant gap persists between this legal entitlement and actual utilization (5).

114: Please harmonize the citation style here

Figure1: WHO Framework of Social Determinants of Health [ (WHO), 2010].

Line 155-156: sentence broken after the word student on line 155.

Lines 167-168: Tables and Figures are meant to be introduced in the manuscript in chronological order and once a Table is introduced, the same should be inserted before a new paragraph but there, Table 3 is mentioned even before Table 1 and Table 3 itself is not inserted until several pages later. Suggest describing what table 3 entails or how it brings out the thick description and delete the phrase “Table 3” from this sentence.

Thick descriptions of the context and participants are provided in the Findings section and Table 3 to enhance transferability.

Line 273 to 279 are repeated in Lines 281 to 287. Please delete one of these two paragraphs.

Please harmonize formatting: avoid mixing alphabetical letters, numerical, hyphens/dash and colons.

221 **A: Structural Determinants of Health;** 300 **B- Intermediate determinants of health;** 360 **3- Cross-cutting Factors**
