# Supplementary material for: Social determinants of access to reproductive health services among Afghan migrant women in Iran: A qualitative study
Source: PLOS Glob Public Health. 2026 Oct 15;6(10):e0007406. doi: 10.1371/journal.pgph.0007406 (PMC13671505; doi:10.1371/journal.pgph.0007406)
Supplement: Supplementary file 15 [file pgph.0007406.s010.docx]

# Response to Reviewer Comments

*Manuscript: Social Determinants of Access to Reproductive Health Services among Afghan Migrant Women in Iran: A Qualitative Study*

We thank the editor for the careful review of the revised manuscript. Below we address each comment point by point.

| **No.** | **Location** | **Reviewer Comment** | **Response / Action Taken** | **Status** |
| --- | --- | --- | --- | --- |
| 1 | Lines 73–79 (Introduction) | This sentence is very long. | The single long sentence describing the SRH status of Afghan migrant women and the relevant legal provisions has been divided into three shorter, clearer sentences: one introducing the intersection of determinants, one describing the legal provisions, and one stating the gap between legal entitlement and actual utilization. | Addressed |
| 2 | Line 114 (Figure 1 caption) | Please harmonize the citation style here. | The citation format "[(WHO), 2010]" has been replaced with the numbered in-text citation style used throughout the manuscript: "Figure 1. WHO framework of social determinants of health (11)." A full manuscript check confirms no remaining name–year citations. | Addressed |
| 3 | Lines 155–156 (Data Processing and Analysis) | Sentence broken after the word "student" on line 155. | The sentence has been corrected and now reads as a complete sentence: "…an Afghan midwifery student served as a peer facilitator to build trust and ensure accurate interpretation." | Addressed |
| 4 | Lines 167–168 (Trustworthiness) | Tables/Figures must be introduced in chronological order; Table 3 was cited before Table 1 and was not inserted until several pages later. Suggest describing what Table 3 entails instead, and delete the phrase "Table 3." | The phrase "and Table 3" has been removed from the sentence, which now reads: "Thick descriptions of the context and participants are provided in the Findings section to enhance transferability." NOTE: in the process of resolving this, we found that Tables 1–3 (and the Figure 1 image) are not currently embedded anywhere in the manuscript file — please re-insert them in chronological order before resubmission (see cover note). | Partially addressed – action needed |
| 5 | Lines 273–279 vs. 281–287 (Findings) | This paragraph is repeated twice; please delete one instance. | The manuscript was checked in full for duplicate paragraphs; the repeated paragraph is no longer present, and no other duplications were found. | Addressed |
| 6 | Findings section headings (Structural / Intermediary / Cross-cutting Factors and subheadings) | Please harmonize formatting: avoid mixing alphabetical letters, numerals, hyphens/dashes and colons (e.g., "221 A:", "300 B-", "360 3-"). | All top-level section headings now use a consistent numeral + period format ("1. Structural Determinants of Health", "2. Intermediary Determinants of Health", "3. Cross-cutting Factors"), and all subheadings use the matching decimal format ("2.1", "2.2", "3.1", "3.2"), with no alphabetical labels, dashes, or colons remaining. | Addressed |

**Outstanding item for the authors:** Tables 1–3 and the Figure 1 image are referenced in the text but are not currently embedded in the manuscript file. These should be inserted in chronological order (Table 1 before Table 2, before Table 3; each immediately following its first mention) prior to resubmission.
