# Supplementary material for: Social determinants of access to reproductive health services among Afghan migrant women in Iran: A qualitative study
Source: PLOS Glob Public Health. 2026 Oct 15;6(10):e0007406. doi: 10.1371/journal.pgph.0007406 (PMC13671505; doi:10.1371/journal.pgph.0007406)
Supplement: Peer review history

# Peer review: Decision Letter 0

**DOI:** 10.1371/journal.pgph.0007406.r001

**Authors:** Maria del Mar Pastor Bravo

18 Mar 2026

PGPH-D-26-00223

Social Determinants of Access to Reproductive Health Services among Afghan Migrant Women in Iran: A Qualitative Study

PLOS Global Public Health

Dear authors,

Thank you for submitting your manuscript to PLOS Global Public Health. After careful consideration, we feel that it has merit but does not fully meet PLOS Global Public Health’s publication criteria as it currently stands. Therefore, we invite you to submit a revised version of the manuscript that addresses the points raised during the review process.

Please submit your revised manuscript by May 02 2026 11:59PM. If you will need more time than this to complete your revisions, please reply to this message or contact the journal office at globalpubhealth@plos.org. When you're ready to submit your revision, log on to https://www.editorialmanager.com/pgph/ and select the 'Submissions Needing Revision' folder to locate your manuscript file.

Please include the following items when submitting your revised manuscript:

- A letter that responds to each point raised by the editor and reviewer(s). You should upload this letter as a separate file labeled 'Response to Reviewers'.
- A marked-up copy of your manuscript that highlights changes made to the original version. You should upload this as a separate file labeled 'Revised Manuscript with Track Changes'.
- An unmarked version of your revised paper without tracked changes. You should upload this as a separate file labeled 'Manuscript'.

Guidelines for resubmitting your figure files are available below the reviewer comments at the end of this letter.

We look forward to receiving your revised manuscript.

Kind regards,

Maria del Mar Pastor Bravo, Ph.D.

Academic Editor

PLOS Global Public Health

**Journal Requirements:**

1. Please ensure that your Ethics Statement is available in its entirety at the beginning of your Methods section, under a subheading 'Ethics Statement'. It must include:

i) The name(s) of the Institutional Review Board(s) or Ethics Committee(s)

ii) The approval number(s), or a statement that approval was granted by the named board(s)

iii) (for human participants or donors) - A statement that formal consent was obtained (must state whether verbal/written) OR the reason consent was not obtained (e.g., anonymity).

2. We note that you have indicated that there are restrictions to data sharing for this study. For studies involving human research participant data or other sensitive data, we encourage authors to share de-identified or anonymized data. However, when data cannot be publicly shared for ethical reasons, we allow authors to make their data sets available upon request. For information on unacceptable data access restrictions, please see http://journals.plos.org/plosone/s/data-availability#loc-unacceptable-data-access-restrictions.

Before we proceed with your manuscript, please address the following prompts:

a) If there are ethical or legal restrictions on sharing a de-identified data set, please explain them in detail (e.g., data contain potentially identifying or sensitive patient information, data are owned by a third-party organization, etc.) and who has imposed them (e.g., a Research Ethics Committee or Institutional Review Board, etc.). Please also provide contact information for a data access committee, ethics committee, or other institutional body to which data requests may be sent.

b) If there are no restrictions, please upload the minimal anonymized data set necessary to replicate your study findings to a stable, public repository and provide us with the relevant URLs, DOIs, or accession numbers. Please see http://www.bmj.com/content/340/bmj.c181.long for guidelines on how to de-identify and prepare clinical data for publication. For a list of recommended repositories, please see https://journals.plos.org/plosone/s/recommended-repositories. You also have the option of uploading the data as Supporting Information files, but we would recommend depositing data directly to a data repository if possible.

Please update your Data Availability statement in the submission form accordingly.

If the reviewer comments include a recommendation to cite specific previously published works, please review and evaluate these publications to determine whether they are relevant and should be cited. There is no requirement to cite these works unless the editor has indicated otherwise.

**Additional Editor Comments (if provided):**

[Note: HTML markup is below. Please do not edit.]

Reviewers' comments:

Reviewer's Responses to Questions

**Comments to the Author**

1. Does this manuscript meet PLOS Global Public Health’s publication criteria?

Reviewer #1: Yes

Reviewer #2: Yes

**********

2. Has the statistical analysis been performed appropriately and rigorously?

Reviewer #1: N/A

Reviewer #2: N/A

**********

3. Have the authors made all data underlying the findings in their manuscript fully available (please refer to the Data Availability Statement at the start of the manuscript PDF file)?

The PLOS Data policy

Reviewer #1: Yes

Reviewer #2: No

**********

4. Is the manuscript presented in an intelligible fashion and written in standard English?

Reviewer #1: Yes

Reviewer #2: Yes

**********

**Reviewer #1:**  As such it is a qualitative study with very limited data as only 20 women and 10 Key informants were included. The qualitative data at best can form the basis for a more detailed study in a larger group of women migrating from Afghanistan. An area not covered in the study is the ethnic background of women from Afghanistan itself. Depending on where they come from for example, Hazra community or from Tajik or Pashtun or Uzhbek, the response and behavior to seek RH varies. This has not been mentioned by the authors. I would also suggest that they authors note clearly how they themselves would use the analysis and what kind of future studies will be planned on their sites. Additional analysis in terms of variation by age, ethinicity, reasons for migration (such as marriage into a Iranian family vs. refugee or undocumented status), prior knowledge and care seeking behaviour when they were in Afghanistan are areas that I hope will be covered in a larger, broader study in future.

**Reviewer #2:**  I have attached a document containing comprehensive feedback and suggestions for the authors' consideration. I hope that these are useful and happy to clarify anything should the authors find this necessary. Thanks.

**********

what does this mean?). If published, this will include your full peer review and any attached files.

**Do you want your identity to be public for this peer review?**  If you choose “no”, your identity will remain anonymous but your review may still be made public.

For information about this choice, including consent withdrawal, please see our Privacy Policy

Reviewer #1: **Yes:**  Lakshmi N Balaji

Reviewer #2: **Yes:**  Dr. Camlus Odhus

**********

[NOTE: If reviewer comments were submitted as an attachment file, they will be attached to this email and accessible via the submission site. Please log into your account, locate the manuscript record, and check for the action link "View Attachments". If this link does not appear, there are no attachment files.]

**Figure Resubmissions:**

After uploading your figures to PLOS’s NAAS tool - https://ngplosjournals.pagemajik.ai/artanalysis, NAAS will process the files provided and display the results in the "Uploaded Files" section of the page as the processing is complete. If the uploaded figures meet our requirements (or NAAS is able to fix the files to meet our requirements), the figure will be marked as "fixed" above. If NAAS is unable to fix the files, a red "failed" label will appear above. When NAAS has confirmed that the figure files meet our requirements, please download the file via the download option, and include these NAAS processed figure files when submitting your revised manuscript.

Attachment Submitted filename: Review comments PGPH-D-26-00223.docx

# Author comment: Author response to Decision Letter 1

**DOI:** 10.1371/journal.pgph.0007406.r002

20 Apr 2026

Attachment Submitted filename: Response to Reviewer PLOS Global Public Health.docx

# Peer review: Decision Letter 1

**DOI:** 10.1371/journal.pgph.0007406.r003

**Authors:** Somayeh Hessam

1 Jun 2026

PGPH-D-26-00223R1

Social Determinants of Access to Reproductive Health Services among Afghan Migrant Women in Iran: A Qualitative Study

PLOS Global Public Health

Dear Dr. Bayat Jozani,

Thank you for submitting your manuscript to PLOS Global Public Health. After careful consideration, we feel that it has merit but does not fully meet PLOS Global Public Health’s publication criteria as it currently stands. Therefore, we invite you to submit a revised version of the manuscript that addresses the points raised during the review process.

Please submit your revised manuscript by Jul 01 2026 11:59PM. If you will need more time than this to complete your revisions, please reply to this message or contact the journal office at globalpubhealth@plos.org. When you're ready to submit your revision, log on to https://www.editorialmanager.com/pgph/ and select the 'Submissions Needing Revision' folder to locate your manuscript file.

Please include the following items when submitting your revised manuscript:

- A letter that responds to each point raised by the editor and reviewer(s). You should upload this letter as a separate file labeled 'Response to Reviewers'.
- A marked-up copy of your manuscript that highlights changes made to the original version. You should upload this as a separate file labeled 'Revised Manuscript with Track Changes'.
- An unmarked version of your revised paper without tracked changes. You should upload this as a separate file labeled 'Manuscript'.

Guidelines for resubmitting your figure files are available below the reviewer comments at the end of this letter.

As the corresponding author, your ORCID iD is verified in the submission system and will appear in the published article. PLOS supports the use of ORCID, and we encourage all coauthors to register for an ORCID iD and use it as well. Please encourage your coauthors to verify their ORCID iD within the submission system before final acceptance, as unverified ORCID iDs will not appear in the published article. *Only*  the individual author can complete the verification step; PLOS staff *cannot*  verify ORCID iDs on behalf of authors.

We look forward to receiving your revised manuscript.

Kind regards,

Somayeh Hessam

Academic Editor

PLOS Global Public Health

**Journal Requirements:**

If the reviewer comments include a recommendation to cite specific previously published works, please review and evaluate these publications to determine whether they are relevant and should be cited. There is no requirement to cite these works unless the editor has indicated otherwise.

Please review your reference list to ensure that it is complete and correct. If you have cited papers that have been retracted, please include the rationale for doing so in the manuscript text, or remove these references and replace them with relevant current references. Any changes to the reference list should be mentioned in the rebuttal letter that accompanies your revised manuscript. If you need to cite a retracted article, indicate the article’s retracted status in the References list and also include a citation and full reference for the retraction notice.

**Additional Editor Comments (if provided):**

[Note: HTML markup is below. Please do not edit.]

Reviewers' comments:

Reviewer's Responses to Questions

**Comments to the Author**

Reviewer #1: All comments have been addressed

Reviewer #2: All comments have been addressed

**********

publication criteria?

Reviewer #1: Yes

Reviewer #2: Yes

**********

3. Has the statistical analysis been performed appropriately and rigorously?

Reviewer #1: N/A

Reviewer #2: N/A

**********

4. Have the authors made all data underlying the findings in their manuscript fully available (please refer to the Data Availability Statement at the start of the manuscript PDF file)?

The PLOS Data policy

Reviewer #1: Yes

Reviewer #2: Yes

**********

5. Is the manuscript presented in an intelligible fashion and written in standard English?

Reviewer #1: Yes

Reviewer #2: Yes

**********

**Reviewer #1:**  While the authors have responded to the second reviewer point by point, they have completely ignored my comments. I had made the following comments and i suggest in both Discussions and conclusions, the authors specifically address these areas:

1. An area not covered in the study is the ethnic background of women from Afghanistan itself. Depending on where they come from for example, Hazra community or from Tajik or Pashtun or Uzhbek, the response and behavior to seek RH varies. This has not been mentioned by the authors.

2. I would also suggest that they authors note clearly how they themselves would use the analysis and what kind of future studies will be planned on their sites. Additional analysis in terms of variation by age, ethinicity, reasons for migration (such as marriage into a Iranian family vs. refugee or undocumented status), prior knowledge and care seeking behaviour when they were in Afghanistan are areas that I hope will be covered in a larger, broader study in future.

The authors need to clearly note how they plan to use this small study further.

**Reviewer #2:**  no further comments. I thank the authors for their meticulous revision.

**********

what does this mean?). If published, this will include your full peer review and any attached files.

**Do you want your identity to be public for this peer review?**  If you choose “no”, your identity will remain anonymous but your review may still be made public.

For information about this choice, including consent withdrawal, please see our Privacy Policy

Reviewer #1: **Yes:**  Lakshmi Narasimhan Balaji

Reviewer #2: **Yes:**  Dr. Camlus Odhus

**********

[NOTE: If reviewer comments were submitted as an attachment file, they will be attached to this email and accessible via the submission site. Please log into your account, locate the manuscript record, and check for the action link "View Attachments". If this link does not appear, there are no attachment files.]

**Figure Resubmissions:**

# Author comment: Author response to Decision Letter 2

**DOI:** 10.1371/journal.pgph.0007406.r004

29 Jun 2026

Attachment Submitted filename: point by point comment.docx

# Peer review: Decision Letter 2

**DOI:** 10.1371/journal.pgph.0007406.r005

**Authors:** Somayeh Hessam

21 Jul 2026

PGPH-D-26-00223R2

Social Determinants of Access to Reproductive Health Services among Afghan Migrant Women in Iran: A Qualitative Study

PLOS Global Public Health

Dear Dr. Bayat Jozani,

Thank you for submitting your manuscript to PLOS Global Public Health. After careful consideration, we feel that it has merit but does not fully meet PLOS Global Public Health’s publication criteria as it currently stands. Therefore, we invite you to submit a revised version of the manuscript that addresses the points raised during the review process.

Please submit your revised manuscript by Aug 20 2026 11:59PM. If you will need more time than this to complete your revisions, please reply to this message or contact the journal office at globalpubhealth@plos.org. When you're ready to submit your revision, log on to https://www.editorialmanager.com/pgph/ and select the 'Submissions Needing Revision' folder to locate your manuscript file.

Please include the following items when submitting your revised manuscript:

- A letter that responds to each point raised by the editor and reviewer(s). You should upload this letter as a separate file labeled 'Response to Reviewers'.
- A marked-up copy of your manuscript that highlights changes made to the original version. You should upload this as a separate file labeled 'Revised Manuscript with Track Changes'.
- An unmarked version of your revised paper without tracked changes. You should upload this as a separate file labeled 'Manuscript'.

Guidelines for resubmitting your figure files are available below the reviewer comments at the end of this letter.

As the corresponding author, your ORCID iD is verified in the submission system and will appear in the published article. PLOS supports the use of ORCID, and we encourage all coauthors to register for an ORCID iD and use it as well. Please encourage your coauthors to verify their ORCID iD within the submission system before final acceptance, as unverified ORCID iDs will not appear in the published article. *Only*  the individual author can complete the verification step; PLOS staff *cannot*  verify ORCID iDs on behalf of authors.

We look forward to receiving your revised manuscript.

Kind regards,

Somayeh Hessam

Academic Editor

PLOS Global Public Health

Journal Requirements:

If the reviewer comments include a recommendation to cite specific previously published works, please review and evaluate these publications to determine whether they are relevant and should be cited. There is no requirement to cite these works unless the editor has indicated otherwise.

Please review your reference list to ensure that it is complete and correct. If you have cited papers that have been retracted, please include the rationale for doing so in the manuscript text, or remove these references and replace them with relevant current references. Any changes to the reference list should be mentioned in the rebuttal letter that accompanies your revised manuscript. If you need to cite a retracted article, indicate the article’s retracted status in the References list and also include a citation and full reference for the retraction notice.

Additional Editor Comments (if provided):

[Note: HTML markup is below. Please do not edit.]

Reviewers' comments:

Reviewer's Responses to Questions

**Comments to the Author**

Reviewer #2: (No Response)

Reviewer #3: All comments have been addressed

**********

publication criteria?

Reviewer #2: Yes

Reviewer #3: Yes

**********

3. Has the statistical analysis been performed appropriately and rigorously?

Reviewer #2: N/A

Reviewer #3: Yes

**********

4. Have the authors made all data underlying the findings in their manuscript fully available (please refer to the Data Availability Statement at the start of the manuscript PDF file)?

The PLOS Data policy

Reviewer #2: No

Reviewer #3: Yes

**********

5. Is the manuscript presented in an intelligible fashion and written in standard English?

Reviewer #2: Yes

Reviewer #3: Yes

**********

Reviewer #2: Dear Authors,

Thanks for submitting a revised manuscript which reads very well. Please find some comments to help strengthen and improve it below.

Lines 17-52: Abstract. Too long, with 382 words. Please shorten to within 300 words.

Lines 39-40. Reference is made to women’s autonomy. While this is appropriate, I wondered whether “agency” might be a better descriptor, considering what the analysis uncovered.

Lines 43-44. Migrant specific or Migrant sensitive? I would recommend ‘migrant sensitive” since the study concludes by stating that considerable policies and actions have already been taken towards integrating Afghan migrants into the health system.

Line 46. Reference is made to a “triad of vulnerability” but this is not mentioned anywhere in the body of the manuscript. Is the author referring to “being a woman, being a migrant, and being subject to a patriarchal society?” If yes (or no), these should be discussed in the body of the manuscript (findings, discussion, conclusion) and the relevant intersectional connections made before being included in the abstract.

Line 51. The sentence has “enhance service equity, reduce health disparity...” These two are essentially the same thing. Omit or replace one to limit repetition/redundancy.

Lines 53-54. Key words. Consider adding “health equity” or “health disparities”, considering the study focus.

Line 73. Replace year 2013 with appropriate citation style.

Line 87. Delete “is based in” and replace with “use”

Line 90. Replace capital T in “This study” with lowercase t.

Line 93. Delete “conversely’ and replace with more suitable conjunctive verb eg “Additionally,...”

Line 103-104. Already used “unique” hence no need to include “idiosyncrasies”/ Instead, you could just replace idiosyncrasies with “determinants”.

Line 110. Consider adding, “...using an intersectionality lens” to the study aim.

Line 111. Insert “health” between reproductive and services to read “reproductive health services”.

General comment: consider using uniform font size within paragraphs.

Lines 154-160. Rigour and trustworthiness. Rather than just mention in passing the various elements of rigour/trustworthiness, please explain exactly what you did.

Lines 161-167. Research team and reflexivity. Clinical understanding is emphasised although the researcher did not interact with participants in their role as clinical health worker (midwife). Consider a more balanced reflection on the researcher’s positionality and implications for the research.

Line 195. Table 3. Consider further refinements as suggested below (ultimately your decision as the researcher)

Macro-economic policy: 3- High cost of health services seems misplaced here.

Social class: 3- job. Could this be better coded as “employment” or “occupation”

Public policy: codes are overlapping. Consider further refinements. Also, these included codes do not seem to speak to “public policy” as a sub-category. Were there better examples of public policies eg Mandatory vaccinations, social or low-cost housing policies, subsidising health care, sin taxes, service integration etc that would better fit here? Also, negative findings or absence of such public policies that would support migrants would still be a key finding.

Material circumstances: Codes need refining. Eg housing quality and nutrition appears in 2 separate codes. Could these be combined or made distinct?

Accessibility and health service delivery: Capitalise H in health.

Consider codes for different elements of access: Financial access (cost/affordability); Distance (Geographical access); and Informational Access (eg health literacy, awareness; various codes under public policy would fit here).

Quality health services and cultural safety: Consider whether analysis could better bring out various facets of quality of care: Timeliness, Safety, Effectiveness (evidence-based care), Patient-centeredness or Responsiveness of SRH services, Service integration and Comprehensiveness, and Service efficiency.

I hope these are useful.

Thanks

Reviewer #3: This manuscript is timely, policy-relevant, and well aligned with the aims of PLOS Global Public Health. The qualitative design is generally appropriate, and the manuscript demonstrates several methodological strengths. However, several issues remain regarding methodological transparency, analytical depth, and interpretation of findings before the manuscript can be considered for publication.

1-The manuscript uses directed qualitative content analysis based on the WHO SDH framework. However, the analysis appears largely deductive.The authors should explicitly clarify:

• Were any inductive themes generated outside the SDH framework?

• Were any data inconsistent with the framework?

• How were new concepts handled during coding?

Otherwise the study risks appearing as merely classifying data into predetermined categories instead of generating qualitative insight.

2-The manuscript states that interviews continued until saturation.

However, it does not explain:

• how saturation was assessed;

• who determined saturation;

• whether code saturation or meaning saturation was achieved;

• after which interview saturation occurred.

A more transparent description consistent with current qualitative reporting standards is recommended

3-The Discussion occupies a disproportionately large part of the manuscript.

Several paragraphs mainly summarize previous literature rather than interpreting the present findings.

Greater emphasis should be placed on:

• what this study uniquely contributes;

• how findings extend previous knowledge;

• implications for policy.

**********

what does this mean?). If published, this will include your full peer review and any attached files.

**Do you want your identity to be public for this peer review?**  If you choose “no”, your identity will remain anonymous but your review may still be made public.

For information about this choice, including consent withdrawal, please see our Privacy Policy

Reviewer #2: **Yes:**  Dr. Camlus Odhus

Reviewer #3: No

**********

[NOTE: If reviewer comments were submitted as an attachment file, they will be attached to this email and accessible via the submission site. Please log into your account, locate the manuscript record, and check for the action link "View Attachments". If this link does not appear, there are no attachment files.]

# Author comment: Author response to Decision Letter 3

**DOI:** 10.1371/journal.pgph.0007406.r006

8 Aug 2026

Attachment Submitted filename: response to rev.docx

# Peer review: Decision Letter 3

**DOI:** 10.1371/journal.pgph.0007406.r007

**Authors:** Somayeh Hessam

24 Aug 2026

PGPH-D-26-00223R3

Social Determinants of Access to Reproductive Health Services among Afghan Migrant Women in Iran: A Qualitative Study

PLOS Global Public Health

Dear Dr. Bayat Jozani,

Thank you for submitting your manuscript to PLOS Global Public Health. After careful consideration, we feel that it has merit but does not fully meet PLOS Global Public Health’s publication criteria as it currently stands. Therefore, we invite you to submit a revised version of the manuscript that addresses the points raised during the review process.

Please submit your revised manuscript by Oct 23 2026 11:59PM. If you will need more time than this to complete your revisions, please reply to this message or contact the journal office at globalpubhealth@plos.org. When you're ready to submit your revision, log on to https://www.editorialmanager.com/pgph/ and select the 'Submissions Needing Revision' folder to locate your manuscript file.

Please include the following items when submitting your revised manuscript:

- A letter that responds to each point raised by the editor and reviewer(s). You should upload this letter as a separate file labeled 'Response to Reviewers'.
- A marked-up copy of your manuscript that highlights changes made to the original version. You should upload this as a separate file labeled 'Revised Manuscript with Track Changes'.
- An unmarked version of your revised paper without tracked changes. You should upload this as a separate file labeled 'Manuscript'.

Guidelines for resubmitting your figure files are available below the reviewer comments at the end of this letter.

As the corresponding author, your ORCID iD is verified in the submission system and will appear in the published article. PLOS supports the use of ORCID, and we encourage all coauthors to register for an ORCID iD and use it as well. Please encourage your coauthors to verify their ORCID iD within the submission system before final acceptance, as unverified ORCID iDs will not appear in the published article. *Only*  the individual author can complete the verification step; PLOS staff *cannot*  verify ORCID iDs on behalf of authors.

We look forward to receiving your revised manuscript.

Kind regards,

Somayeh Hessam

Academic Editor

PLOS Global Public Health

Journal Requirements:

If the reviewer comments include a recommendation to cite specific previously published works, please review and evaluate these publications to determine whether they are relevant and should be cited. There is no requirement to cite these works unless the editor has indicated otherwise.

Additional Editor Comments (if provided):

[Note: HTML markup is below. Please do not edit.]

Reviewers' comments:

Reviewer's Responses to Questions

**Comments to the Author**

Reviewer #2: All comments have been addressed

Reviewer #3: All comments have been addressed

**********

publication criteria?

Reviewer #2: Yes

Reviewer #3: Yes

**********

3. Has the statistical analysis been performed appropriately and rigorously?

Reviewer #2: N/A

Reviewer #3: Yes

**********

4. Have the authors made all data underlying the findings in their manuscript fully available (please refer to the Data Availability Statement at the start of the manuscript PDF file)?

The PLOS Data policy

Reviewer #2: No

Reviewer #3: Yes

**********

5. Is the manuscript presented in an intelligible fashion and written in standard English?

Reviewer #2: Yes

Reviewer #3: Yes

**********

Reviewer #2: I have left a few comments in the attached document. Once these are addressed, the manuscript should be good to go

Reviewer #3: The article is fully relevant to the scope of the journal in terms of its topic, and its strengths include the use of WHO-SDH, maximum diversity sampling, interviews with migrant women and key informants, and the use of COREQ.However, in the R3 version there are still problems with methodological clarity, coherence of the theoretical framework, depth of qualitative analysis, length of Discussion, contradictions and repetitions of the text, and some overly strong claims

1. The most important flaw: The distinction between WHO-SDH and Intersectionality is still not entirely clear

he authors state in the Abstract and Introduction that the study was conducted with the WHO-SDH framework using an intersectionality lens, but it is not clear exactly how intersectionality was operationalized.

Add a separate paragraph in the Methods section that specifies:

exactly what role intersectionality played in the sampling;

how it was incorporated into the coding and interpretation;

what intersections were specifically analyzed;

whether intersectionality was an independent theoretical framework or merely an interpretive lens.

Also, in the Discussion, claims such as “interact multiplicatively rather than additively” should be used with caution, since the study is qualitative and the data did not measure interaction in a quantitative sense.

2. The “first study” claim should be made more cautious

3. There is a serious problem with the reporting of the study time

The Abstract states:

“Data collection took place in Tehran from April 2024 over six months”

But the Methods state:

“From April 2024 to June 2025”

4. Trustworthiness is still duplicated

The Methods section contains almost the same thing about Trustworthiness twice

5-5-Discussion is still too long

6- Some claims about healthcare costs are higher than the study data

The authors state in several places that eliminating out-of-pocket costs will result in:

“long-term cost-saving”

and a reduction in high-risk deliveries and communicable diseases.

This may make sense from a policy perspective, but was not measured in this study.

7-Table 3 needs a conceptual revision

Table 3 is too broad and some codes are not at the same conceptual level.

**********

what does this mean?). If published, this will include your full peer review and any attached files.

**Do you want your identity to be public for this peer review?**  If you choose “no”, your identity will remain anonymous but your review may still be made public.

For information about this choice, including consent withdrawal, please see our Privacy Policy

Reviewer #2: **Yes:**  Dr Camlus Odhus

Reviewer #3: No

**********

[NOTE: If reviewer comments were submitted as an attachment file, they will be attached to this email and accessible via the submission site. Please log into your account, locate the manuscript record, and check for the action link "View Attachments". If this link does not appear, there are no attachment files.]

Attachment Submitted filename: Reviewer feedback.docx

# Author comment: Author response to Decision Letter 4

**DOI:** 10.1371/journal.pgph.0007406.r008

27 Aug 2026

Attachment Submitted filename: PLOS Global Public Health point to point.docx

# Peer review: Decision Letter 4

**DOI:** 10.1371/journal.pgph.0007406.r009

**Authors:** Somayeh Hessam

7 Sep 2026

PGPH-D-26-00223R4

Social Determinants of Access to Reproductive Health Services among Afghan Migrant Women in Iran: A Qualitative Study

PLOS Global Public Health

Dear Dr. Bayat Jozani,

Thank you for submitting your manuscript to PLOS Global Public Health. After careful consideration, we feel that it has merit but does not fully meet PLOS Global Public Health’s publication criteria as it currently stands. Therefore, we invite you to submit a revised version of the manuscript that addresses the points raised during the review process.

Please submit your revised manuscript by Oct 07 2026 11:59PM. If you will need more time than this to complete your revisions, please reply to this message or contact the journal office at globalpubhealth@plos.org. When you're ready to submit your revision, log on to https://www.editorialmanager.com/pgph/ and select the 'Submissions Needing Revision' folder to locate your manuscript file.

Please include the following items when submitting your revised manuscript:

- A letter that responds to each point raised by the editor and reviewer(s). You should upload this letter as a separate file labeled 'Response to Reviewers'.
- A marked-up copy of your manuscript that highlights changes made to the original version. You should upload this as a separate file labeled 'Revised Manuscript with Track Changes'.
- An unmarked version of your revised paper without tracked changes. You should upload this as a separate file labeled 'Manuscript'.

Guidelines for resubmitting your figure files are available below the reviewer comments at the end of this letter.

As the corresponding author, your ORCID iD is verified in the submission system and will appear in the published article. PLOS supports the use of ORCID, and we encourage all coauthors to register for an ORCID iD and use it as well. Please encourage your coauthors to verify their ORCID iD within the submission system before final acceptance, as unverified ORCID iDs will not appear in the published article. *Only*  the individual author can complete the verification step; PLOS staff *cannot*  verify ORCID iDs on behalf of authors.

We look forward to receiving your revised manuscript.

Kind regards,

Somayeh Hessam

Academic Editor

PLOS Global Public Health

Journal Requirements:

If the reviewer comments include a recommendation to cite specific previously published works, please review and evaluate these publications to determine whether they are relevant and should be cited. There is no requirement to cite these works unless the editor has indicated otherwise.

Please review your reference list to ensure that it is complete and correct. If you have cited papers that have been retracted, please include the rationale for doing so in the manuscript text, or remove these references and replace them with relevant current references. Any changes to the reference list should be mentioned in the rebuttal letter that accompanies your revised manuscript. If you need to cite a retracted article, indicate the article’s retracted status in the References list and also include a citation and full reference for the retraction notice.

Additional Editor Comments (if provided):

[Note: HTML markup is below. Please do not edit.]

Reviewers' comments:

Reviewer's Responses to Questions

**Comments to the Author**

Reviewer #2: All comments have been addressed

Reviewer #3: All comments have been addressed

**********

publication criteria?

Reviewer #2: Yes

Reviewer #3: Yes

**********

3. Has the statistical analysis been performed appropriately and rigorously?

Reviewer #2: N/A

Reviewer #3: Yes

**********

4. Have the authors made all data underlying the findings in their manuscript fully available (please refer to the Data Availability Statement at the start of the manuscript PDF file)?

The PLOS Data policy

Reviewer #2: Yes

Reviewer #3: Yes

**********

5. Is the manuscript presented in an intelligible fashion and written in standard English?

Reviewer #2: Yes

Reviewer #3: Yes

**********

**Review Comments to the Author**

Reviewer #2: Requesting a few minor editorial changes e.g., removal of a duplicated paragraph.

Reviewer #3: In terms of topic, relevance, and relevance to PLOS Global Public Health, the article is publishable and of scientific value. However, in the current version, the main problem is no longer “lack of content”; rather, it is over-interpretation, several methodological/numerical inconsistencies, and claims that go beyond qualitative data.

1-Serious discrepancy in data collection time

In the Abstract it says:

“Data collection took place in Tehran from April 2024 over six months”

But in the Methods it says:

“From April 2024 to June 2025”

2-The most important theoretical flaw: WHO-SDH and intersectionality are by no means fully operationalized. The paper states:

"Using the WHO-SDH framework"and then:"Using an intersectional perspective"

But the methods do not specify exactly how intersectionality is incorporated into the analysis.

3-Reduce causal claims .At several points in the article, words are used that are too causal for qualitative data.

4-The Discussion is long. Although the authors have reduced the number of headings, the Discussion is still effectively re-divided into several subtopics.

5-Data Availability Statement needs serious revision

6-Results should be more evidence-based and less interpretive.

**********

what does this mean?). If published, this will include your full peer review and any attached files.

**Do you want your identity to be public for this peer review?**  If you choose “no”, your identity will remain anonymous but your review may still be made public.

For information about this choice, including consent withdrawal, please see our Privacy Policy

Reviewer #2: **Yes:**  Dr. Camlus Odhus

Reviewer #3: No

**********

[NOTE: If reviewer comments were submitted as an attachment file, they will be attached to this email and accessible via the submission site. Please log into your account, locate the manuscript record, and check for the action link "View Attachments". If this link does not appear, there are no attachment files.]

Attachment Submitted filename: Review comments.docx

# Author comment: Author response to Decision Letter 5

**DOI:** 10.1371/journal.pgph.0007406.r010

14 Sep 2026

Attachment Submitted filename: Response_to_Reviewer_PGPH-D-26-00223R4 (1).docx

# Editor report: Decision Letter 5

**DOI:** 10.1371/journal.pgph.0007406.r011

**Authors:** Somayeh Hessam

16 Sep 2026

Social Determinants of Access to Reproductive Health Services among Afghan Migrant Women in Iran: A Qualitative Study

PGPH-D-26-00223R5

Dear PhD student Bayat Jozani,

We are pleased to inform you that your manuscript 'Social Determinants of Access to Reproductive Health Services among Afghan Migrant Women in Iran: A Qualitative Study' has been provisionally accepted for publication in PLOS Global Public Health.

Before your manuscript can be formally accepted you will need to complete some formatting changes, which you will receive in a follow up email. A member of our team will be in touch with a set of requests.

Please note that your manuscript will not be scheduled for publication until you have made the required changes, so a swift response is appreciated.

IMPORTANT: The editorial review process is now complete. PLOS will only permit corrections to spelling, formatting or significant scientific errors from this point onwards. Requests for major changes, or any which affect the scientific understanding of your work, will cause delays to the publication date of your manuscript.

If your institution or institutions have a press office, please notify them about your upcoming paper to help maximize its impact. If they'll be preparing press materials, please inform our press team as soon as possible -- no later than 48 hours after receiving the formal acceptance. Your manuscript will remain under strict press embargo until 2 pm Eastern Time on the date of publication. For more information, please contact globalpubhealth@plos.org.

Thank you again for supporting Open Access publishing; we are looking forward to publishing your work in PLOS Global Public Health.

Best regards,

Somayeh Hessam

Academic Editor

PLOS Global Public Health

***********************************************************

Reviewer Comments (if any, and for reference):
